# Compact 9dBEs Enable Efficient and Precise Genome Editing in Mammalian Cells and In Vivo

**DOI:** 10.1002/advs.77279

**Published:** 2026-08-19

**Authors:** Qingquan Xiao, Zhijin Tian, Luqi Weng, Kun Xu, Dingyi Han, Zhaowei Wu, Junnan Li, Shangpu Li, Qijing Zhang, Xiaohui Zhao, Guoling Li, Yinghui Wei, Zhen Gu

**Affiliations:** ^1^ State Key Laboratory of Advanced Drug Delivery and Release Systems Jinhua Institute of Zhejiang University School of Pharmacy Zhejiang University Hangzhou China; ^2^ International Joint Agriculture Research Center for Animal Bio‐Breeding of Ministry of Agriculture and Rural Affairs College of Animal Science and Technology Northwest A&F University Yangling China; ^3^ Hainan Institute Northwest A&F University Sanya China; ^4^ Center for Excellence in Brain Science and Intelligence Technology (Institute of Neuroscience) Chinese Academy of Sciences Shanghai China; ^5^ School of Physical Science and Technology ShanghaiTech University Shanghai China; ^6^ School of Pharmacy Hangzhou Medical College Hangzhou China; ^7^ Zhongshan Institute for Drug Discovery Shanghai Institute of Materia Medica Chinese Academy of Sciences Zhongshan China

**Keywords:** base editor, CRISPR‐Cas9d, gene editing, Pcsk9 gene, Tyr gene

## Abstract

Despite the promise of DNA base editors for diverse genome editing applications, their utility remains constrained by off‐target effects, which are exacerbated by short spacers in miniature systems and the large size of Cas9‐derived editors, which impedes adeno‐associated virus (AAV) delivery. Here, guided by structural insights into a compact Cas9d nuclease from *Deltaproteobacteria*, we developed an efficient Cas9d system (Cas9d^Ultra^) through gRNA and protein engineering, and further developed its base editors (9dBEs). Cas9d^Ultra^ and 9dBEs enabled efficient and precise genome editing in human cells. Notably, 9dCBE induced premature termination codons in 89% of mouse pups by microinjection, facilitating robust disease modeling. Furthermore, a single AAV delivering 9dCBE achieved efficient *Pcsk9* editing and a concomitant reduction in serum LDL‐C levels in mice. Collectively, this study establishes a series of compact, potent genome editing tools poised to advance biological and biomedical translational research.

## Graphical abstract

As a compact type II‐D system, the Cas9d‐based platform holds great potential for in vivo applications. Through rational engineering, its derived base editors (9dBEs) enable efficient disease modeling while facilitating single‐vector AAV delivery for in vivo genome editing. These miniature tools offer a robust strategy for basic research and biomedical translation.

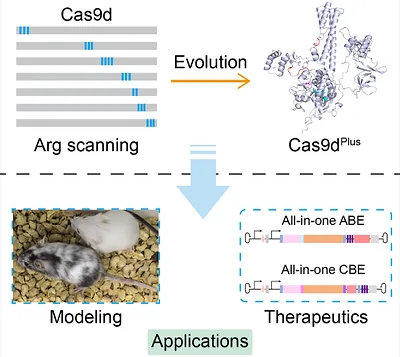

## 1 Introduction

DNA base editors (BEs) are promising tools for correcting or disrupting genes by introducing base conversion in the genome [1, 2]. Key advantages of BEs are their high editing efficiency without requiring DNA double‐strand break (DSB), DNA templates and cell division [1, 2]. However, the large size of Cas9 and its derivatives poses challenges for delivery using a single adeno‐associated virus (AAV) vector, the most commonly used vehicle for in vivo gene editing, which has a cargo capacity typically restricted to approximately 4.7 kb [3]. Moreover, base editing efficiency is highly dependent on enzymes possessing an HNH nuclease activity. Among the well‐characterized CRISPR systems, type II Cas9 protein, consisting of three RuvC‐like domains and one HNH‐like endonuclease domain [4, 5], has been used to develop base editors [1, 2, 6, 7, 8, 9, 10] and prime editors (PEs) [11].

The relatively large size of *Sp*Cas9 systems has motivated efforts to evolve smaller Cas orthologs or develop alternative systems. Among these alternatives, including *Slug*Cas9 (1,054 aa) [12], *Nme2*Cas9 (1,082 aa) [13], TnpB [14, 15], Cas12f [16] and Fanzor [17, 18], only IscB (∼500 aa) [19] possesses both an HNH domain and a compact size. Although previous studies by us and others have successfully engineered *Ogeu*IscB, IscB.m16 and DelIscB, to develop miniature DNA editors, their short spacers (14‐16 nt) and restricted targeting scope present obvious limitations [20, 21, 22, 23, 24, 25, 26]. These constraints result in increased potential off‐target sites in IscB‐derived tools and many inaccessible pathogenic loci due to insufficient targeting range. Therefore, engineering compact genome editing tools with a broad targeting scope, high efficiency and precision remains crucial for advancing both research and therapeutic applications.

A potential platform emerged with the discovery of MG34‐1 Cas9d system [27] (hereafter referred to as Cas9d), a 747‐amino acid (aa) type II‐D Cas9 ortholog from *Deltaproteobacteria*. Representing an evolutionary bridge between IscB and larger Cas9 systems, this protein uniquely combines a compact size with a standard 20‐nt spacer. For DNA targeting, Cas9d requires a specific NGG protospacer adjacent motif (PAM) at the 3’ end of the target DNA for guide RNA (gRNA) binding. The folded gRNA acts as a structural scaffold interacting with the small, flexible REC domain to form a functional recognition module achieving DNA targeting [28, 29]. These advantages make Cas9d highly amenable to being developed into genome modification tools. However, although demonstrating staggered dsDNA cleavage activity in vitro and in *E. coli* cells, and lacking collateral ssDNA cleavage activity, wild‐type Cas9d has shown no detectable nuclease activity in mammalian cells [27], and thus severely limit its application.

In this study, to address these limitations, we strategically engineered the Cas9d nuclease by leveraging structural insights from the predicted Cas9d‐sgRNA‐dsDNA ternary complex and available cryo‐EM data [28, 29]. Through iterative rounds of protein engineering and gRNA optimization, we developed a robust Cas9d^Ultra^ system, which comprised four residue substitutions (V281R/G492R/M544R/D582R) and an optimized gRNA‐v3. By fusing the Cas9d^Ultra^ nickase with different deaminases, we further developed the Cas9d^Ultra^‐derived adenine base editor (9dABE) and cytosine base editor (9dCBE). Both 9dBEs exhibited robust base editing across various endogenous loci. We validated the efficiency of Cas9d^Ultra^ and 9dCBE by efficiently generating *Tyr*‐disrupted mouse models via embryo microinjection. Furthermore, intraperitoneal (IP) delivery of 9dBEs using a single AAV vector achieved efficient base editing at the *Pcsk9* locus, significantly reducing serum low‐density lipoprotein cholesterol (LDL‐C) levels in mice. Collectively, our study provides a set of compact, high‐efficiency CRISPR‐Cas9d systems and base editors, offering a versatile toolkit for both fundamental research and therapeutic applications.

## 2 Results

### 2.1 Structure‐Guided Engineering of the gRNA Scaffold

**FIGURE 1:**
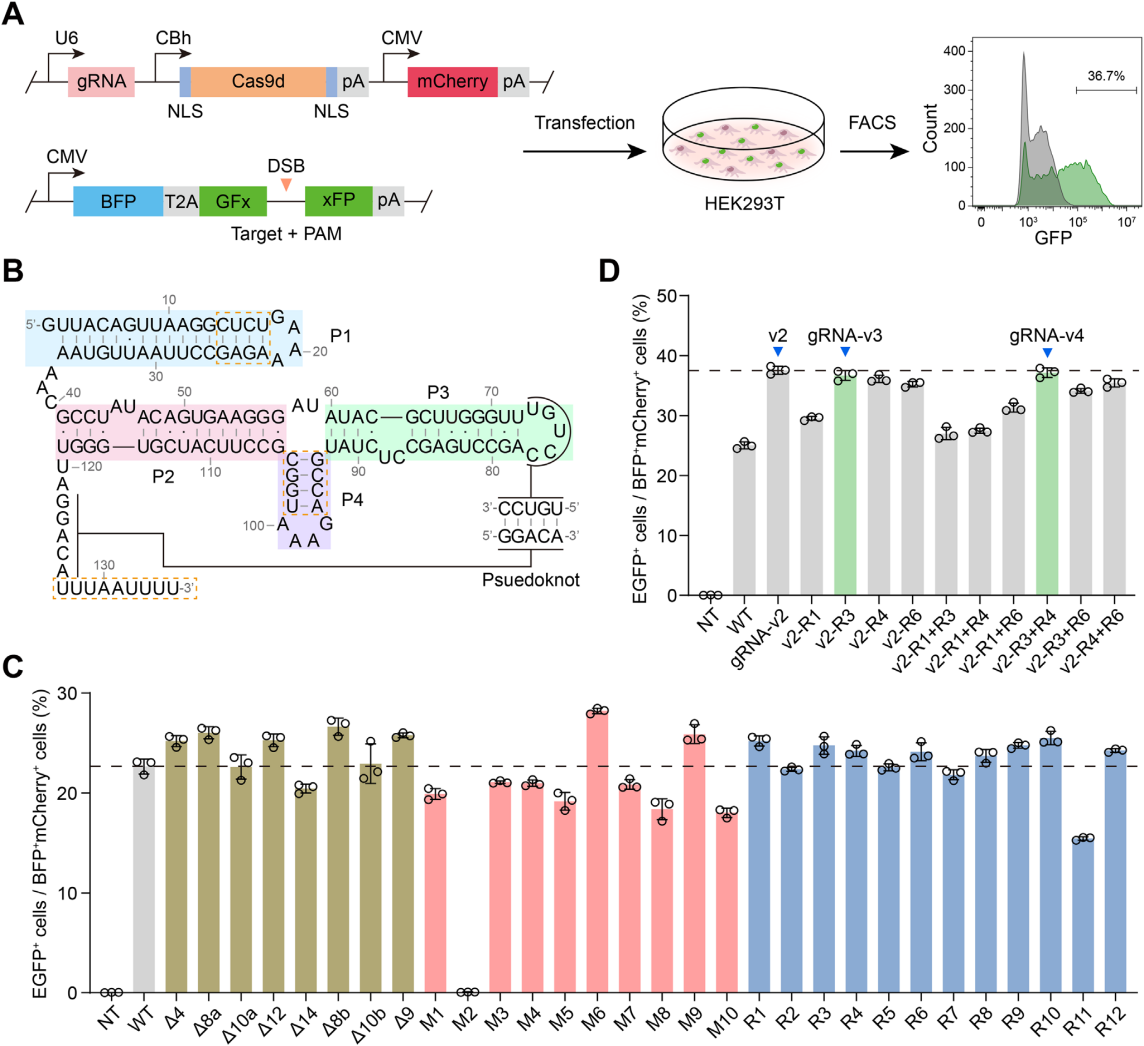
Engineered gRNA scaffold enhances Cas9d cleavage activity and reduces size. (A) Schematic of the GFxxFP reporter system used to evaluate Cas9d‐mediated DNA cleavage in HEK293T cells. (B) Predicted secondary structure of the Cas9d gRNA scaffold. P1‐P4 denote distinct stem‐loops. Dashed boxes indicate the truncated regions. (C) Editing efficiency in reporter system of gRNA variants with scaffold truncations, substitutions or mutations. The dashed line indicates the wild‐type editing level. M represents the substitution of U with C at G·U mismatch sites. R denotes the replacement of A:U or U:A with G:C base pairs. (D) Combination of beneficial mutations generating the optimized scaffolds gRNA‐v2/v3/v4. Data are presented as mean ± standard deviation (s.d.). (n  =  3 independent biological replicates).

The type II‐D CRISPR effector protein, Cas9d, is characterized by its compact size of approximately 750 aa (Figure S1). Despite its reported in vitro activity, a previous study suggested that Cas9d fails to directly cleave the human genome when being delivered via mRNA [27]. To date, whether MG34‐1 Cas9d can be engineered for mammalian genome editing remains unknown. To sensitively detect Cas9d‐mediated cleavage in mammalian cells, we applied a fluorescence reporter system (GFxxFP) previously developed in our laboratory [20, 21], which comprises a blue fluorescent protein (BFP) and an activatable enhanced green fluorescent protein (EGFP) (Figure 1A). EGFP activation is triggered through the single‐strand annealing repair pathway following Cas9d cleavage of the target sequence. Upon co‐expressing human‐codon‐optimized Cas9d and its cognate gRNA with the GFxxFP reporter in HEK293T cells, fluorescence‐activated cell sorting (FACS) assay performed 48 h post‐transfection revealed that approximately 20% of the BFP^+^mCherry^+^ cells were EGFP‐positive (Figure S2A). This result confirmed that Cas9d is indeed functional in mammalian cells and serves as a viable candidate for further engineering.

Guide RNA engineering is an extensively used strategy to improve the performance of programmable endonucleases [16, 20, 21, 22, 23, 24, 30]. To elucidate the structural basis of Cas9d‐gRNA interactions, we predicted the 3D structure of the Cas9d‐gRNA‐dsDNA ternary complex using AlphaFold3 [31]. The gRNA architecture contains four main parts, assigned as P1‐P4, specifically four stems and three main loops (Figure 1B, Figure S2B,C). Notably, a loop within the P3 forms a pseudoknot by base pairing with five bases at the gRNA 3’ tail, further stabilizing the ribonucleoprotein complex (Figure 1B, Figure S2B,C). To optimize gRNA compatibility and stability, we performed comprehensive engineering, including truncating base pairs in the P1 and P4 stems and nucleotides 127–135 (nt); substituting mismatched U at G·U wobble sites with C to create G:C pairs; and independently replacing A:U or U:A base pairs with G:C base pairs. Several variants showed improved cleavage activity (Figure 1C). Through iterative combination of these beneficial mutations, we developed gRNA‐v0, featuring an 8‐nt deletion in both P1 and P4, removal of a 9‐nt 3’ tail, and an M6 (67U>C) mutation, which enhanced activity while reducing length by 25‐nt (Figure S2A). We further refined this scaffold by substituting the 73U:126A pair in the pseudoknot with 73G:126C to produce gRNA‐v1; subsequent incorporation of the M9 (112U>C) mutation yielded gRNA‐v2, which exhibited superior editing efficiency (Figure S2D). Furthermore, incorporation of the R3 (4G:35C) or combined R3+R4 (4G:35C + 6G:33C) mutations yielded variants gRNA‐v3 and gRNA‐v4, respectively. These variants demonstrated increased thermostability while maintaining the high editing efficiency of gRNA‐v2 (Figure 1D). Collectively, these results demonstrate that our optimized gRNA scaffold improves Cas9d activity while reducing its size by 25 nt.

### 2.2 Protein Engineering Improves Cas9d Nuclease Efficiency

**FIGURE 2:**
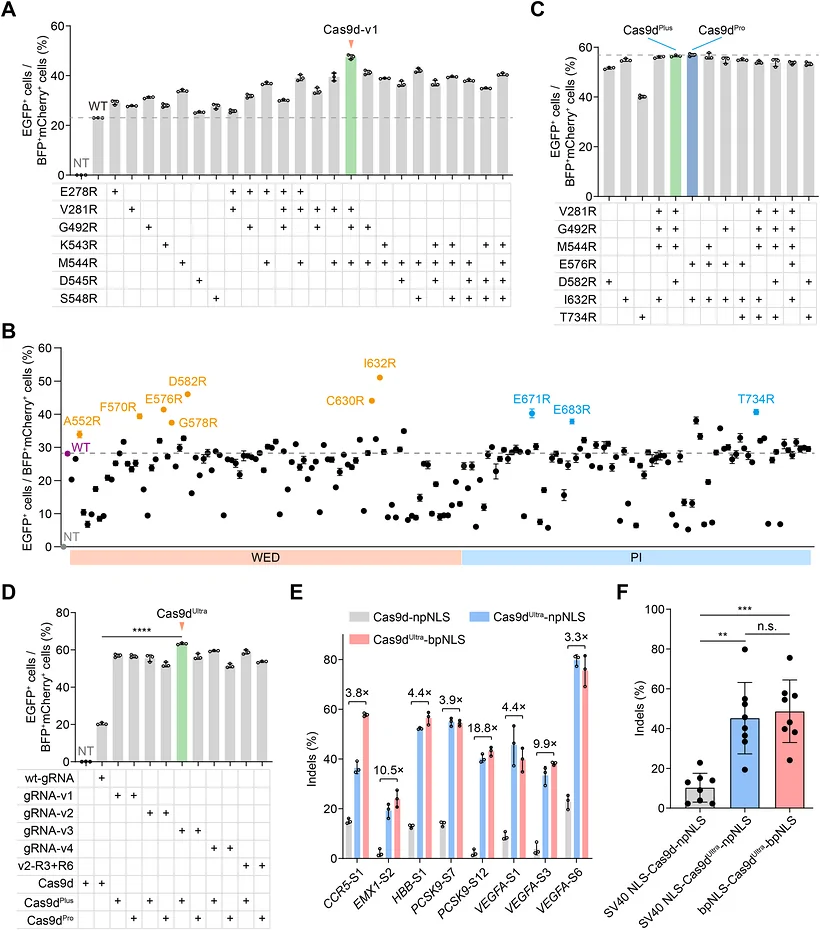
Engineered Cas9d protein enhances nuclease activity in mammalian cells. (A) Combination of beneficial mutations within the RuvC domains increased cleavage efficiency. The Cas9d‐v1 variant (V281R/G492R/M544R) exhibited the highest efficiency among all variants. (B) Single arginine substitutions within the WED and PI domains enhanced Cas9d efficiency. (C) Combination of mutations across the RuvC, WED and PI domains further augmented cleavage activity at the GFxxFP reporter. (D) The Cas9d^Ultra^ system (Cas9d^Plus^ + gRNA‐v3) showed maximal cleavage efficiency among all Cas9d‐gRNA combinations. An unpaired two‐tailed Student's *t*‐test was used to compare the means. ****P < 0.0001. (E) Cleavage efficiency of Cas9d and Cas9d^Ultra^ systems fused with different nuclear localization signals (NLSs) at eight endogenous sites. (bpNLS: bipartite NLS; npNLS: nucleoplasmin NLS). (F) Statistical analysis of the cleavage efficiency data presented in (E). Data are represented as mean ± s.d. Statistical significance was assessed using one‐way analysis of variance (ANOVA) followed by Dunnett's T3 post‐hoc test for multiple comparisons. **P < 0.01; ***p < 0.001. n.s., not significant. (n  =  3 independent biological replicates in (A) to (E)).

Previous studies by us and others have demonstrated that substitution of amino acids with positively charged residues, typically arginine (Arg, R), could enhance the interactions between the Cas effector and gRNA‐target DNA [16, 20, 21, 22, 23, 32]. To improve the activity of Cas9d, we developed a co‐expression system (Figure S3A) that ensures consistent use of gRNA and reporter dosage across all Cas9d variants. Given the essential role of the RuvC domain in catalytic activity, we first performed Arg‐screening across its three RuvC domains. Several single Arg substations, including E278R, V281R, G492R, K543R, M544R, D545R and S548R showed enhanced cleavage efficiency (Figure S3B). By combining these beneficial mutations, we generated a high‐activity variant consisting of V281R/G492R/M544R substitutions (named Cas9d‐v1) (Figure 2A).

Inspired by the study that engineering the wedge (WED) domain can further boost nuclease performance [33], and considering that the WED and PAM‐interacting (PI) domains cooperatively govern PAM recognition, we extended Arg‐scanning to the WED and PI domains to explore potential synergistic effects. A variety of single mutations were observed showing improved activity, particularly I632R achieving a cleavage efficiency of 51.06% ± 0.03% (Figure 2B). Further combination of these mutations from the first two rounds of optimization generated two superior variants, one containing V281R/G492R/M544R/D582R (referred to as Cas9d^Plus^) and the other containing E576R/I632R (termed Cas9d^Pro^), both of which displayed higher efficiency (Figure 2C, Figure S3C). Since these variants were characterized using wild‐type gRNA scaffold, we next investigated whether their combination with our optimized gRNA variants could further augment editing performance. Using the GFxxFP reporter, we evaluated four gRNA variants with two top‐performing Cas9d mutants. Notably, the gRNA‐v3 coupled with Cas9d^Plus^ (named as the Cas9d^Ultra^ system) demonstrated superior editing efficiency (63.32% ± 0.47%) (Figure 2D), resulting in a 3.1‐fold improvement over the wild‐type Cas9d system (20.44% ± 0.58%).

Given that nuclear localization signals (NLSs) are pivotal for efficient mammalian genome editing [34, 35, 36, 37], we systematically evaluated various NLS configurations for both wild‐type Cas9d and Cas9d^Ultra^ across eight genomic loci in HEK293T cells. Results demonstrated that a dual‐terminal bpNLS configuration exhibited optimal performance compared to conventional NLS architectures (Figure 2E,F). Furthermore, we characterized the PAM requirements of both wild‐type Cas9d and Cas9d^Plus^ using an unbiased bacterial depletion assay (Figure S4A). Enrichment analysis revealed that Cas9d^Plus^ exhibited enhanced prokaryotic activity (Figure S4B,C), while maintaining the canonical NGG PAM recognition (Figure S3D). Consequently, integration of rationally designed gRNA scaffolds, protein directed evolution, and optimized NLS configuration collectively boosted Cas9d nuclease activity in both prokaryotic and eukaryotic systems.

### 2.3 Deployment of the Cas9d^Ultra^ System for Human Genome Editing

**FIGURE 3:**
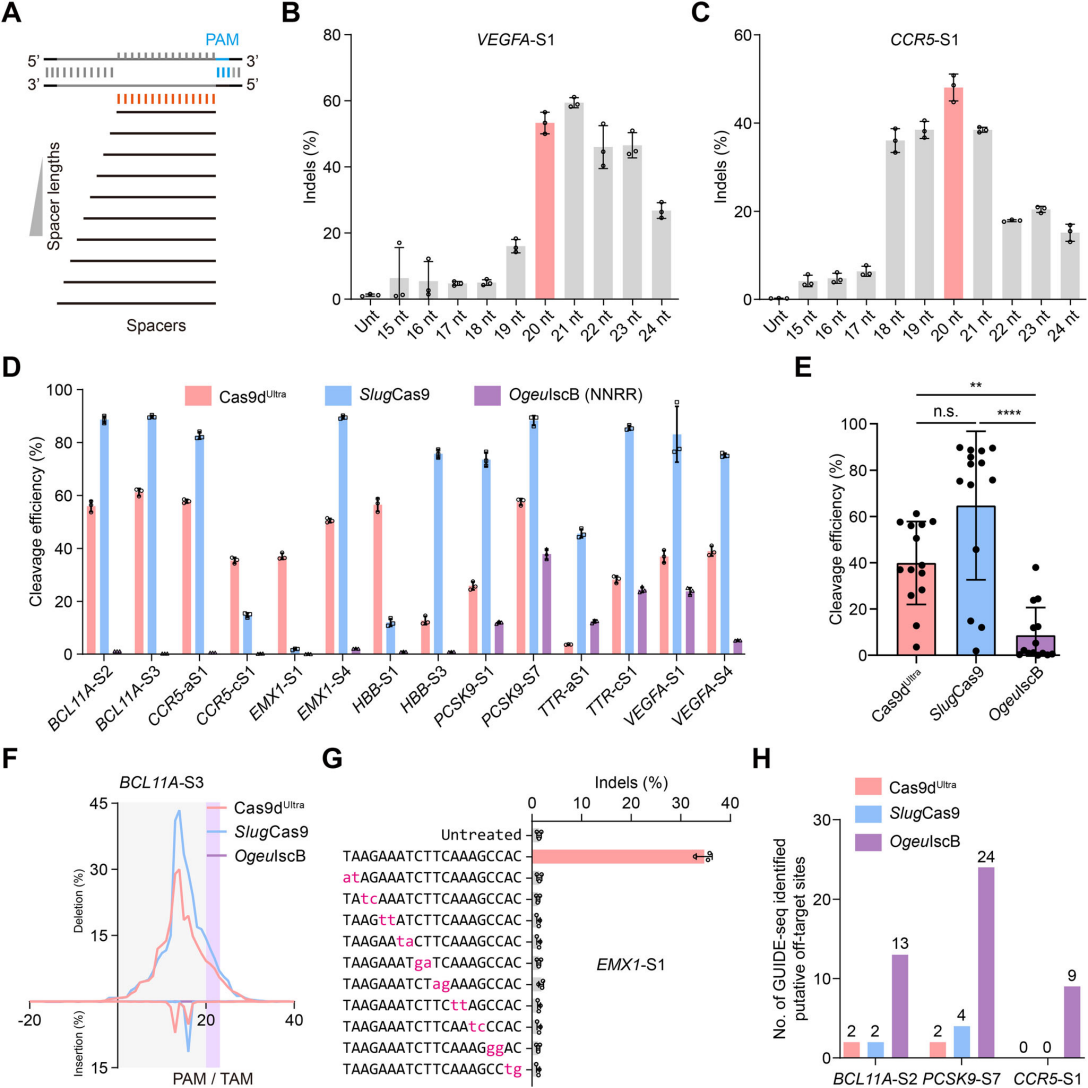
Comprehensive evaluation of the Cas9d^Ultra^ system for genome editing in human cells. (A) Schematic representation of spacers lengths optimization at endogenous target sites. (B, C) Cleavage efficiencies at the *VEGFA*‐S1 (B) and *CCR5*‐S1 (C) loci using gRNAs with different spacer lengths. Unt denotes untreated controls. (D, E) Evaluation of cutting efficiency of Cas9d^Ultra^, *Slug*Cas9 and *Ogeu*IscB at 14 endogenous loci, efficiency of individual sites (D) and collective statistical comparison analysis (E). Statistical analysis was determined by Kruskal‐Wallis test. **P < 0.01; ****p < 0.0001. n.s. denotes non‐significant. (n = 14 sites). (F) Indel profiles of Cas9d^Ultra^, *Slug*Cas9 and *Ogeu*IscB at the *BCL11A*‐S3 locus in HEK293T cells. (G) Assessment of Cas9d^Ultra^ mismatch tolerance at the endogenous *EMX1*‐S1 site. Magenta lowercase letters indicate mismatched bases within the spacer; mismatches significantly impair Cas9d^Ultra^ cleavage activity. (H) Total number of putative off‐target sites detected by GUIDE‐seq for Cas9d^Ultra^, *Slug*Cas9 and *Ogeu*IscB at three genomic targets. Data in (B) to (E) and (G) are presented as mean ± s.d. In (E), each point represents the average editing of n  =  3 independent biological replicates. (n  =  3 independent biological replicates in (B) to (D) and (G)).

While previous in vitro studies indicated that Cas9d can cleave DNA using 17–20 nt spacers [27, 29], the optimal spacer length required for efficient endogenous genome editing remains undefined. To address this, we systematically investigated different spacer lengths targeting three endogenous sites (*VEGFA*‐S1, *CCR5*‐S1 and *PCSK9*‐S7) in HEK293T cells (Figure 3A). Our results demonstrated that Cas9d^Ultra^ requires a minimal spacer length of 17 nt for effective genomic DNA cleavage (Figure S5A). Notably, 20‐nt spacers consistently exhibited high editing efficiency across all these sites (Figure 3B,C, Figure S5A), suggesting 20 nt as the optimal spacer length for this system. We further evaluated the performance of Cas9d^Ultra^ against *Slug*Cas9 [12, 38] and *Ogeu*IscB (NNRR variant [22]) at 14 endogenous human sites (Table S4). Cas9d^Ultra^ showed significantly superior editing efficiency compared to *Ogeu*IscB, while maintaining competitive, albeit slightly lower, efficiency relative to *Slug*Cas9 (Figure 3D,E). Analysis of the indel profiles at *BCL11A*‐S3, *HBB*‐S1 and *EMX1*‐S4 sites revealed that Cas9d^Ultra^ primarily generates deletions centered at position 15 (PAM positions 21–23), with a deletion window spanning from ‐1 to 26 (Figure 3F, Figure S5B).

Since Cas9 tolerates up to 3 mismatches between gRNA and genomic DNA [39, 40], this raises concerns about off‐target effects. To verify the mismatch discrimination of Cas9d^Ultra^, we examined consecutive double mismatches along the spacer at two endogenous sites (*EMX1*‐S1 and *HBB*‐S1) in HEK293T cells. Cas9d^Ultra^ demonstrated minimal tolerance for these mismatches, suggesting high stringency (Figure 3G, Figure S5C). To assess genome‐wide specificity in an unbiased manner, we performed GUIDE‐seq assay at the *PCSK9*‐S7, *BCL11A*‐S2 and *CCR5*‐S1 sites. Notably, Cas9d^Ultra^ induced off‐target editing at only two sites per locus for *PCSK9*‐S7 and *BCL11A*‐S2, markedly fewer than those identified in *Slug*Cas9 (2 and 4 sites) and *Ogeu*IscB (13 and 24 sites; Figure 3H, Figure S6). Furthermore, at the *CCR5*‐S1 site, no off‐target events were detected for Cas9d^Ultra^ and *Slug*Cas9, whereas *Ogeu*IscB generated 9 off‐target edits. Collectively, these findings establish Cas9d^Ultra^ as a highly efficient and high‐fidelity system for precision genome editing.

### 2.4 Cas9d^Ultra^‐Derived 9dBEs Enable Efficient Base Editing

Given that nicking the non‐edited strand improves base editing efficiency in mammalian cells [2], we engineered Cas9d^Ultra^ nickases by substituting the conserved catalytic residues—Asp10 in the RuvC domain and/or His375 in the HNH domain—with alanine. Using dual‐target reporter systems with 15‐bp or 20‐bp insertions [20, 21], we validated that Cas9d^Ultra^ nickases exhibited high nicking activity at both reporters (Figure S7A). This indicates that Cas9d^Ultra^ is a promising candidate to being developed into base editors. To generate Cas9d^Ultra^‐derived adenine and cytosine base editors, we fused an evolved, high‐fidelity TadA8e^V106W^ variant [41] to the N‐terminus of Cas9d^Ultra‐D10A^, resulting in 9dABE (Figure S7B). Similarly, by appending an optimized human APOBEC3A^W104A^ [42, 43] and an uracil DNA glycosylase inhibitor (UGI) to Cas9d^Ultra‐D10A^, we generated 9dCBE (Figure S7B).

**FIGURE 4:**
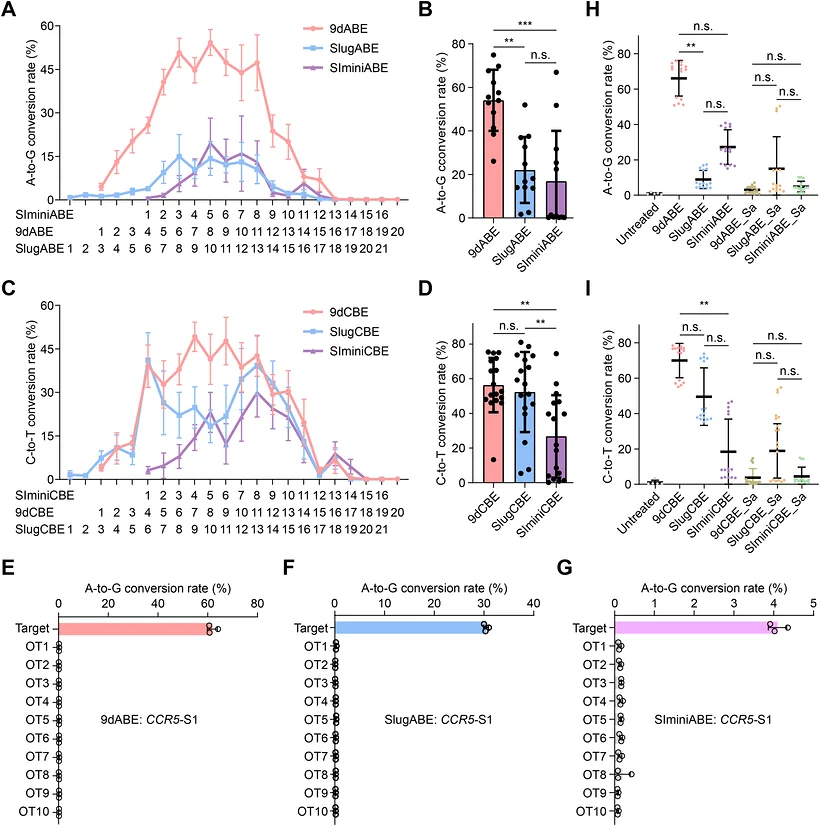
Performance and specificity of 9dBEs in human cells. (A, C) Line charts depicting the editing windows and position‐specific average base editing efficiency for ABEs (A) and CBEs (C). Values are the average editing efficiency at each target A/C position, derived from three independent biological replicates across 12 (ABEs) and 17 (CBE) endogenous sites. Data are shown as the mean ± s.e.m. (B, D) Summary histograms showing the base editing efficiencies for ABE (B) and CBE (D) at endogenous sites. Data are collected from 12 (ABEs) and 17 (CBEs) loci, respectively, and presented as means ± s.d. Each point represents the average optimal base editing efficiency at a specific genomic site of three independent biological replicates. (E to G) Assessment of gRNA‐dependent off‐target effects for 9dABE (E), SlugABE (F) and SIminiABE (G) at the *CCR5*‐S1 locus. Data are displayed as mean ± s.d. (H, I) Evaluation of gRNA‐independent off‐target editing for ABEs (H) and CBEs (I) across 3 endogenous sites and 5 R‐loop regions. Data are presented as mean ± s.d. Each dot represents the average optimal base editing efficiency at each endogenous site of three independent biological replicates. To compare the means, Kruskal‐Wallis test was used in (B, D, H and I). **P < 0.01; ***p < 0.001. n.s. denotes non‐significant.

To systematically characterize the editing profiles of 9dBEs, we designed dozens of PAM/TAM‐matched endogenous loci to compare their performance with SlugBEs [38] and SIminiBEs [22] (Figure S7C, Tables S5 and S6). Although all ABEs exhibited an effective editing window of 5–7 bp, 9dABE showed the highest efficiency across this range, with an optimal window of 6 bp (Figure 4A). Across 12 tested sites, 9dABE achieved significantly higher average A‐to‐G editing efficiency than its counterparts (9dABE: 54.08% ± 14.04% vs. SlugABE: 22.07% ± 15.13% and SIminiABE: 16.86% ± 23.20%; Figure 4B, Figures S8A and S9), albeit with a slight increase in indel frequency (Figure S8C). For cytosine editing across 17 endogenous sites, 9dCBE and SlugCBE exhibited similar editing windows, both of them broader than that of SIminiCBE (Figure 4C). Notably, 9dCBE and SlugCBE showed comparable average C‐to‐T editing efficiencies (56.12% ± 15.67% and 52.17% ± 23.00%, respectively), both of which markedly outperformed SIminiCBE (26.67% ± 23.66%; Figure 4D, Figures S8B and S10). In addition, all CBEs induced similar indel frequencies (Figure S8D). Furthermore, parallel assessments at several endogenous loci indicated that both 9dABE and 9dCBE achieved editing efficiencies comparable to those of *Sp*Cas9‐derived base editors (SpBEs) (Figure S11). Together, these results demonstrate that Cad9d^Ultra^‐derived compact base editors offer superior editing performance compared to these smaller systems.

To rigorously evaluate the specificity of the 9dBEs, we assessed both gRNA‐dependent and gRNA‐independent off‐target effects in HEK293T cells, with SlugBEs and SIminiBEs serving as benchmarks. Potential gRNA‐dependent off‐target sites were predicted using Cas‐OFFinder [44] (Tables S11‐S16), while gRNA‐independent off‐target editing was examined via an orthogonal R‐loop assay [45]. Targeted deep sequencing detected only one off‐target event for both 9dABE and 9dCBE (Figure 4E, Figures S12A and S13A). In comparison, SlugABE induced several off‐target edits, although SlugCBE remained highly specific (Figure 4F, Figures S12B and S13B). SIminiABE and SIminiCBE exhibited one and two off‐target sites, respectively (Figure 4G, Figures S12C and S13C). Furthermore, the R‐loop assay revealed that 9dBEs and SIminiBEs displayed comparable, low levels of gRNA‐independent editing, both of which were slightly lower than those of the SlugBEs, which showed elevated off‐target activity at several R‐loop sites (Figure 4H,I). Collectively, these results substantiated that 9dBEs are highly efficient, compact tools possessing superior specificity compared to SlugBEs and SIminiBEs at the tested genomic loci.

### 2.5 Mouse Embryo and In Vivo Genome Editing

**FIGURE 5:**
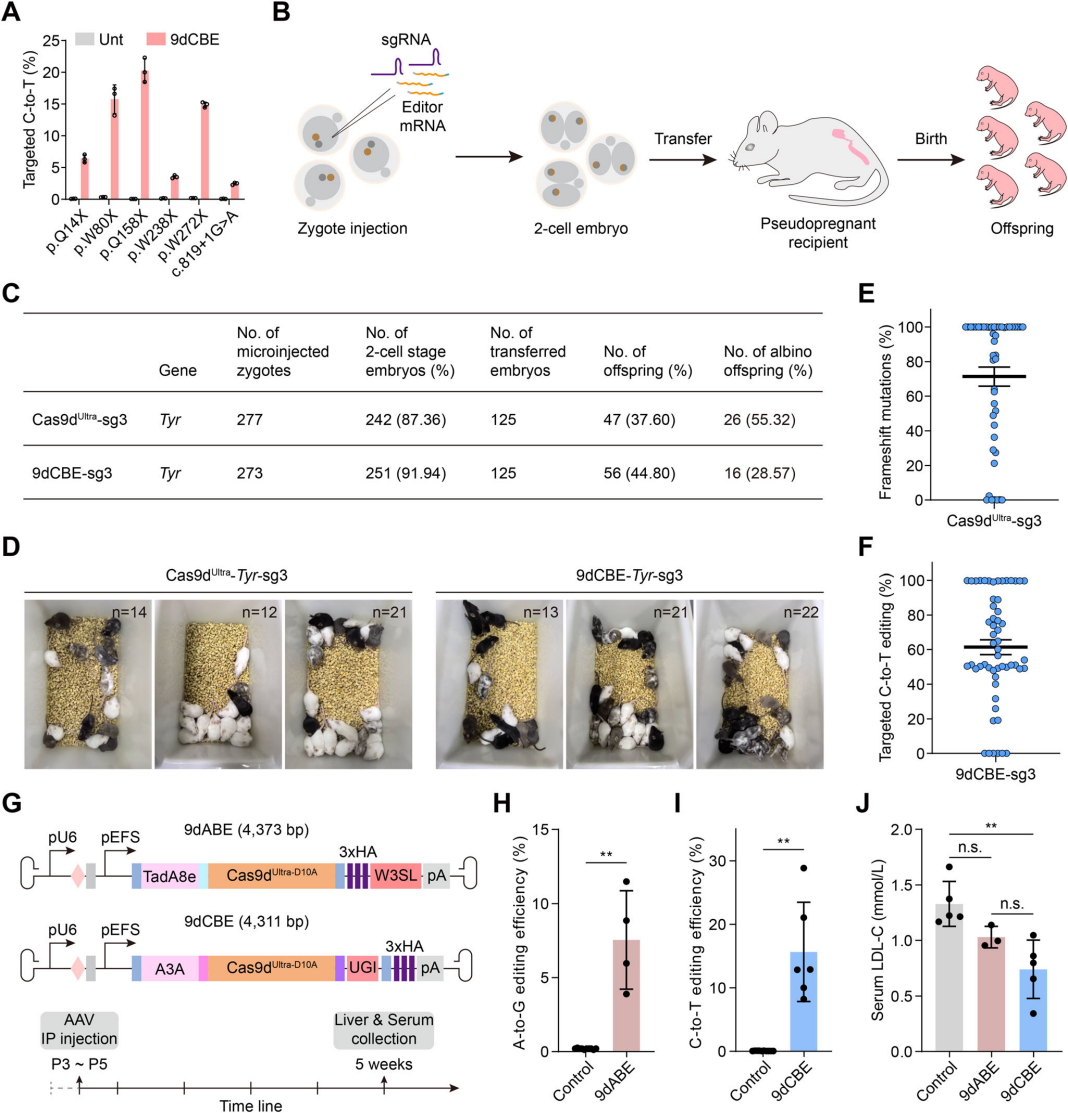
Cas9d^Ultra^ and 9dBEs are efficient platforms for gene editing in mouse embryos and in vivo. (A) Targeted C‐to‐T editing efficiency mediated by 9dCBE at the *Tyr* locus in N2a cells (n = 3 independent biological replicates). (B) Experimental workflow for generating *Tyr*‐modified mouse models by microinjection of Cas9d^Ultra^ or 9dCBE mRNA and sgRNA into zygotes. (C) Summary of embryonic development and F0 mouse productions. (D) Representative fur phenotypes of F0 mice resulting from *Tyr* editing by Cas9d^Ultra^ (n = 47 mice) and 9dCBE (n = 56 mice). Images were captured at postnatal day 15. (E, F) *Tyr* editing efficiencies in F0 mice generated by Cas9d^Ultra^ (E; n = 47 mice) and 9dCBE (F; n = 56 mice). (G) Schematic of single‐AAV‐delivered 9dBEs and the experimental workflow for *in vivo Pcsk9* editing. (H, I) In vivo base editing efficiencies of 9dABE (H) and 9dCBE (I). (J) Reduction of serum LDL‐C levels in treated mice. Each data point represents an individual mouse. Data in (A, and H to J) are presented as mean ± s.d. Data in (E and F) are shown as mean ± s.e.m.

The capacity for efficient genome modification across diverse cell types and in vivo is paramount for the translational utility of CRISPR‐based tools. To evaluate these tools in mouse embryo, we designed six gRNAs targeting exon 1 of the *tyrosinase* (*Tyr*) gene, which encodes a key melanin biosynthesis enzyme [23, 46, 47, 48], and screened the editing efficiency in neuroblastoma (N2a) cells (Figure S14A). Efficiency screening identified sg3 as the most potent candidate for both Cas9d^Ultra^ and 9dCBE (Figure 5A, Figure S14B). Subsequently, we assessed editing efficiency in black‐furred D2B6F1 zygotes by microinjection of sg3 with Cas9d^Ultra^ or 9dCBE mRNA (Figure 5B). High blastocyst developmental rates—80% (8/10) and 93% (13/14), respectively—confirmed the low cytotoxicity of our systems (Figure S14C,D). Targeted deep sequencing revealed high‐efficiency editing, with an average 49.61% frameshift mutations in Cas9d^Ultra^‐injected zygotes and 53.19% C‐to‐T conversion in the 9dCBE group (Figure S14E‐G).

To generate *Tyr*‐disrupted models, we transferred the injected two‐cell embryos into foster mothers, yielding 47 and 56 F0 pups for Cas9d^Ultra^ and 9dCBE, respectively (Figure 5C,D). Phenotypic and genotypic analyses confirmed *Tyr* disruption in 85% (40/47) of Cas9d^Ultra^‐treated pups (71.36% average efficiency) and 89% (50/56) of 9dCBE‐treated pups (Figure 5E,F). Specifically, albino phenotypes in the Cas9d^Ultra^ group predominantly resulted from frameshift deletions, whereas 9dCBE‐generated albino mice were characterized by nearly complete premature termination codons (PTCs) (Figure S15). Importantly, deep sequencing of predicted off‐target sites in fully albino F0 mice (n = 3 per group) detected no off‐target editing, underscoring the high fidelity of these tools in vivo (Figure S16, Table S17).

We further extended this platform to in vivo therapeutic editing by targeting *Pcsk9* (a key regulator of cholesterol metabolism) [49], in the liver via single‐AAV delivery. After screening gRNA candidates in cultured cells (Figure S17A‐C), we selected sg2 (targeting a splice site) for 9dABE and sg5 (inducing a PTC) for 9dCBE. Neonatal C57BL/6 mice (P3 – P5) received a systemic intraperitoneal (IP) injection of 5×10^13^ vg/mouse of the respective single‐AAV vectors. In the fifth week, liver tissues and serum were harvested for molecular and biochemical analyses (Figure 5G). 9dABE achieved editing efficiencies ranging from 3.5% to 11.9% (Figure 5H, Figure S17D), while 9dCBE mediated an average of 15.7% (8.3%‐28.9%) efficiency (Figure 5I, Figure S17E). Crucially, this genomic modification translated into a significant reduction in serum LDL‐C levels compared to PBS‐treated controls (Figure 5J). Collectively, these findings highlight the robust potential of Cas9d^Ultra^ and its derived base editors for precise genome editing in both embryonic and therapeutic in vivo contexts.

## 3 Discussion

RNA‐guided endonucleases are fundamental to prokaryotic biology, serving in antiviral defense and facilitating RNA‐guided transposition by CRISPR‐associated transposases [50, 51, 52]. While CRISPR‐Cas (Cas9, Cas12) [51, 53] and non‐CRISPR nucleases (IscB [19, 21], TnpB [14, 15], Fanzor [17, 18, 47]) have been extensively adapted for genome editing, their broader applications are often hampered by large size, lack of HNH domain, or reliance on a short spacer (14‐16 nt). These limitations present persistent challenges for efficient delivery, feasible application and high‐fidelity targeting. To address these limitations, we focused on the compact (747 aa) type II‐D ortholog MG34‐1 Cas9d, which utilizes a 20‐nt spacer and represents a promising candidate for miniature editing platform [27]. Despite a previous study suggesting undetectable activity in mammalian cells [27], our coordinated engineering of the gRNA scaffold and nuclease largely improved performance in HEK293T cells and enabled development of nickases. Notably, the Cas9d^Ultra^ exhibited superior *Tyr* editing potency compared to previously reported compact nucleases, achieving a 55% (26/47) albino F0 pups rate in mouse embryos, surpassing eIscB‐D (5/12, 42%) [23] and enNlovFz2 (3/9, 33%) [47].

Given our focus on developing highly efficient and precise compact base editors, we developed 9dABE and 9dCBE by tethering deaminases and UGI to the Cas9d nickase. These 9dBEs demonstrated efficiencies superior or comparable to SlugBEs and SIminiBEs. Crucially, the small size of 9dBEs facilitated their packaging into a single AAV vector, enabling effective in vivo therapeutic targeting of the *Pcsk9* gene. Single AAV delivery bypasses the complexity and reduced efficiency associated with dual‐AAV systems, highlighting the translational potential of our platform. Furthermore, Cas9d‐derived BEs possess a broader effective editing window than their counterparts, offering a distinct advantage for applications such as lineage tracing or in vivo barcode generation. However, this expanded window may increase the risk of bystander edits, a critical consideration for precision editing. Future efforts to narrow the editing window and develop customized editing profiles will be essential to facilitate these tools for diverse applications.

Our findings also demonstrated that protein engineering enhances Cas9d activity more efficiently than gRNA optimization. This suggests that the compact architecture of Cas9d may necessitate an extended gRNA scaffold to compensate for inherent structural and functional constraints, explaining why its gRNA is typically longer than those of larger Cas9 orthologs [27, 28, 29, 54]. Consequently, future optimization should prioritize protein‐directed evolution. Although 9dBEs performed robustly, we noted that the cleavage activity of optimized Cas9d^Ultra^ remains slightly lower than that of *Slug*Cas9; the underlying mechanism for this disparity warrants further investigation. Furthermore, we confirmed that while a minimum of 17 nt is required for Cas9d activity [29], a 20‐nt spacer is essential for achieving optimal performance. Since shorter spacers could increase off‐target risks, and engineering key residues in the PAM‐distal region allows for the accommodation of longer spacers [55, 56], this strategy could be adapted to further improve the fidelity of Cas9d derivatives.

Moreover, both wild‐type Cas9d and Cas9d^Plus^ strictly require a canonical NGG PAM, limiting their target scope. Although PAM specificity can be relaxed or altered by direct residue engineering [57] or phage‐assisted evolution [58], current methods like saturation mutagenesis remain labor‐intensive [21]. Transitioning toward unbiased, high‐throughput rational mutagenesis integrated with AI‐based optimization [59, 60] will be essential for the scalable and predictive of Cas9d PAM recognition. Study showed that expanding PAM compatibility often correlates with increased off‐target effects [57]. Thus, striking a balance between targeting scope and high‐fidelity remains a critical challenge. Developing a suite of Cas9d variants with diverse and stringent PAM requirements may ultimately outperform those with broader yet less specific PAM profiles.

## 4 Conclusions

In summary, through structure‐guided rational design, combined with gRNA scaffold optimization and protein engineering, we have generated compact, efficient, and highly specific Cas9^Ultra^ variant and its derived base editors. These tools demonstrated robust editing efficiency in mammalian cells, mouse embryos and *in vivo Pcsk9* therapeutic targeting. By overcoming the single AAV delivery constraints inherent to larger CRISPR systems, this miniature platform offers a versatile toolkit for both basic research and therapeutic applications.

## 5 Materials and Methods

### 5.1 Plasmids Cloning

All PCR amplifications were performed using Phanta Max Super‐Fidelity DNA Polymerase (Vazyme). PCR products were separated by agarose gel electrophoresis and purified using a gel extraction kit (OMEGA Bio‐tek). The human codon‐optimized Cas9d gene and its corresponding gRNA scaffold were synthesized by Tsingke Biotech. Plasmids pQH440 and pTH03 were kindly provided by Hui Yang's laboratory [20, 21]. As for the *Slug*Cas9 and SlugBEs were gifts from Yongming Wang [12], and SIminiBEs and *Ogeu*IscB (NNRR variant) were gifts from Hanhui Ma [22]. The Cas9d coding sequence was cloned into the mammalian cells expression backbone (pQH440) driven by CBh promoter between *Xho*I and *Eco*RI restriction sites using the 2×pEASY basic seamless cloning and assembly kit (TransGen Biotech). The gRNA scaffold was subsequently cloned with *Xba*I and *Kpn*I after Cas9d assembled. The GFxxFP reporter plasmids were adapted from pTH03. For spacer cloning, complementary oligonucleotides were annealed and ligated into the *Bpi*I‐digested pU6‐gRNA_scaffold‐pCBh‐Cas9d‐bGH_polyA expression cassette using T4 DNA ligase (Thermo Fisher Scientific). All oligonucleotides were synthesized by Tsingke Biotech and Genewiz. All colonies were verified from the promoter to poly(A) tail by Sanger sequencing (Tsingke Biotech and Genewiz). All primers and DNA sequences used in this study are listed in Tables S1‐S7.

### 5.2 Cell Culture and Transfection

HEK293T and Neuro‐2a (N2a) cell lines were gifts from Hui Yang's laboratory. Both cell lines were cultured in high glucose DMEM (Gibco) supplemented with 10% FBS (Gibco), 1% penicillin‐streptomycin‐glutamine (Gibco) and 1% NEAA (Gibco) in a humidified incubator at 37°C with 5% CO_2_. To assess the cleavage activity of the Cas9d system and its engineered variants during optimization, HEK293T cells were seeded on poly‐D‐lysine‐coated 48‐well plates (Corning) and transfected at about 70% confluency. Briefly, transfections were performed using polyethylenimine (PEI) with a total of 800 ng plasmid DNA per well, comprising the Cas9d‐associated systems and the GFxxFP reporter system at a 1:1 molar ratio. For endogenous gene editing, transfections were conducted on 24‐well plates. Cells at ∼70% confluency were transfected with 1.6 µg per well of all‐in‐one plasmids encoding the editing systems, using PEI according to the manufacturer's manual.

To efficiently edit the murine *Tyr* gene, six single guide RNAs (sgRNAs) were screened in cultured N2a cells. In brief, N2a cells were seeded in 24‐well plates and transfected at approximately 70% confluency around 12 h after seeding. A total of 1.6 µg of all‐in‐one editor plasmids were transfected per well using Lipofectamine 2000 reagent (Invitrogen), following the manufacturer's instructions.

### 5.3 FACS Analysis and Cell Sorting

To quantify the editing efficiency of Cas9d and its associated variants at the GFxxFP reporter, flow cytometric analysis was performed using a Beckman CytoFlex S instrument to measure GFP fluorescence in BFP^+^mCherry^+^ cells. In brief, after 48 h of transfection, cells were harvested by trypsinization and resuspended in culture medium containing 10% FBS to neutralize the trypsin. Data acquisition with FSC, SSC and fluorescent channels (450, 525, and 561 nm emission, respectively) was stopped once 20,000 events were recorded within the single‐cell gate. Further quantification of editing efficiency was conducted using FlowJo X (v10.0.7) by calculating the percentage of GFP^+^ cells within the BFP^+^mCherry^+^ population. For endogenous editing efficiency assessment, cells were processed identically 48 h post‐transfection. Approximately 15,000 cells from the top 35% mCherry‐positive population were sorted into 500 µL of culture medium in a 1.5 mL tube using a BD FACSAria Fusion Flow Cytometer. Genomic DNA of sorted cells was extracted by lysis with 25 µL of lysis buffer containing proteinase K (Vazyme) per sample, followed by incubation at 55°C for 50 min and 95°C for 10 min. Cell lysates were stored at −20°C for future use.

### 5.4 Targeted Deep Sequencing and Analysis

To assess the editing efficiency at endogenous sites, the genomic regions of interest were amplified from cell lysates by PCR using 2×Taq mix (Vazyme) with barcoded primers. PCR products were separated by agarose gel electrophoresis and target bands were purified using a gel extraction kit (Omega Bio‐tek). For library preparation, pooled DNA fragments underwent end repair, adapter ligation and amplification using the VAHTS Universal Pro DNA Library Prep Kit for Illumina (Vazyme, ND608), according to the manufacturer's protocol. The libraries were subjected to paired‐end sequencing on an Illumina NovaSeq 6000 (Genewiz). Deep sequencing data were first demultiplexed using a custom script based on sample barcodes. Indel frequency and base editing efficiency were analyzed using CRISPResso2 (v 2.3.1, [61]) with the following key parameters: –plot_window_size 22 –window_around_sgRNA 20 –quantification_window_center ‐3 (for cleavage efficiency), and –plot_window_size 16 –window_around_sgRNA 16 –quantification_window_center ‐10 (for base frequencies). All primer sequences used for amplifying the target sites are listed in Table S8.

### 5.5 Guide RNA‐Dependent Off‐Target Assay

To assess base editor specificity, guide RNA‐dependent potential off‐target sites were identified across the human (hg38) or mouse (mm10) reference genome using Cas‐OFFinder (v2.4, [44]). The search parameters allowed ≤ 5 mismatches within the target region and required canonical PAM sequences: NGG for Cas9d and 9dBEs, NNGG for SlugBEs and NNRR for SIminiBEs. For ABEs, potential off‐target sites associated with three on‐target sites (*CCR5*‐S1, *EMX1*‐S1 and *TTR*‐S1) were used for further off‐target effects analysis. Similarly, for CBEs, potential off‐target sites associated with three on‐target sites (*PCSK9*‐S3, *TTR*‐S1, and *VEGFA*‐S4) were analyzed. All validated potential off‐target loci are provided in Tables S11‐S16. Subsequently, the top ten potential off‐target sites for each on‐target site were validated by PCR amplification followed by high‐throughput sequencing (HTS). The HTS data were analyzed using CRISPResso2 (v 2.3.1) with parameters identical to those used for on‐target BE analysis.

### 5.6 Orthogonal R‐Loop Assay

To evaluate Cas‐independent DNA off‐target editing by ABEs and CBEs, as previously described, an orthogonal R‐loop assay [45] was used to assess the ability of BEs to mutate single‐stranded DNA within an off‐target R‐loop region generated by an orthogonal, catalytically inactive *Staphylococcus aureus* Cas9 (d*Sa*Cas9). Briefly, HEK293T cells were seeded in 24‐well plates. At ∼70% confluence, cells were co‐transfected with a total of 1.6 µg of plasmid DNA (comprising both the all‐in‐one BE system and all‐in‐one d*Sa*Cas9 system) using 3.2 µL of PEI, following the manufacturer's protocol. Similar to on‐target experiments, cells with ∼35% GFP and mCherry double‐positive cells were sorted 48 h after transfection. Subsequently, the on‐target site and five related off‐target *Sa*Cas9 R‐loop sites (Table S10) were amplified with 2×Taq mix for deep sequencing and further analysis of off‐target effects.

### 5.7 GUIDE‐seq Assay

GUIDE‐seq assay was conducted as previously described [62]. In brief, HEK293T cells were seeded in 6‐cm dishes (Corning) before transfection. At 70–80% confluency, cells were co‐transfected with 5.5 µg of all‐in‐one editing plasmid, 3.5 µg of puromycin resistance plasmid and 18 pmol of annealed double‐stranded oligodeoxynucleotide (dsODN) using PEI. At 24 h post‐transfection, cells were selected with puromycin at final concentration of 5 µg/mL. Approximately 1×10^7^ viable cells were harvested 72 h after transfection. Genomic DNA was extracted using the CTAB method and sheared to an average size of 500 bp using NEBNext dsDNA Fragmentase (NEB). Libraries were prepared using the VAHTS Universal DNA Library Prep Kit for Illumina V3 (Vazyme) through end‐repair, dA‐tailing and adapter ligation. Fragments containing the dsODN integration were enriched with two rounds of nested PCR using anchored primers. Sequencing was performed on an Illumina NovaSeq 6000 (GentleGen) in paired‐end mode. Data were analyzed using the GUIDE‐seq pipeline (https://github.com/aryeelab/guideseq). The PAM sequences were defined as NGG for Cas9d^Ultra^, NNGG for *Slug*Cas9, and NNRR for the *Ogeu*IscB NNRR‐variant. For all orthologs, the analysis allowed for up to 6 mismatches and applied a read frequency threshold of ≥ 1%. All off‐target sites identified by GUIDE‐seq are provided in Tables S18‐S26.

### 5.8 PAM Library Generation

The PAM library was generated as previously described [21]. In brief, a randomized PAM plasmid library was constructed using synthesized oligonucleotides (HuaGene) containing eight randomized nucleotides downstream of the Cas9d target sequence. The randomized single‐stranded DNA (ssDNA) oligos were converted to double‐stranded DNA (dsDNA) by annealing to a short primer, followed by synthesis of the complementary strand using the DNA polymerase I, large (Klenow) fragment (New England Biolabs). The resulting dsDNA fragment was purified and then assembled into a *Hind*III and *Eco*RI linearized pACYC184 vector backbone using NEBuilder HiFi DNA Assembly (New England Biolabs). The assembly products were purified and electroporated into TransforMax EC100 electrocompetent *E. coli* following the manufacturer's protocol. The transformed cells were plated on a square (25 cm × 25 cm) LB agar plate supplemented with chloramphenicol and incubated at 37°C. After 13 h of incubation, the colonies were collected by scraping and plasmid DNA was extracted using a NucleoBond Xtra Midiprep kit (Machery Nagel).

### 5.9 PAM Depletion Assay and Bioinformatic Analysis

The PAM depletion assay was carried out as described previously [21]. Briefly, 200 ng of PAM library plasmid and 300 ng of all‐in‐one Cas9d system expression plasmid were co‐electroporated into TransforMax EC100 electrocompetent *E. coli*. The transformed cells were recovered in antibiotic‐free SOC medium for 1 h at 37°C to allow expression of antibiotic resistance genes and then selected on a 22 cm × 22 cm LB agar plate containing ampicillin and chloramphenicol, followed by culture at 37°C for 13 h. Subsequently, colonies were collected and plasmid DNA was extracted in the same way as PAM library preparation. The sequences encompassing target and PAM were amplified and subjected to paired‐end (150 bp) sequencing on an Illumina NovaSeq 6000 platform (Genewiz).

Randomized PAM regions were extracted, counted and normalized to the total PAM counts for each sample. For each specific PAM, frequencies occurring more than once were retained and the log fold change (logFC) of these retained PAMs was calculated as the log ratio compared to the non‐target control, with a 1e‐9 pseudocount adjustment [19]. PAM depletions with a logFC < ‐3σ (s.d.) were considered statistically significant and these sequences were collected to build sequence logos using WebLogo (version 3.7.12; [63]).

### 5.10 In Vitro Transcription of Editors’ mRNA and *Tyr*‐gRNA

The editors’ mRNA and mouse *Tyr*‐targeting gRNA were transcribed in vitro as previously described [47]. Briefly, a T7 promoter sequence was added to the 5’ end of the Cas9d^Ultra^, 9dCBE and gRNA transcription templates by PCR amplification. The PCR products were purified using a gel extraction kit (OMEGA Bio‐tek) and used as transcription templates. The mRNA was transcribed using the mMESSAGE mMACHINE T7 Ultra Transcription Kit (Life Technologies) and gRNA was transcribed using the MEGAshortscript T7 Transcription Kit (Life Technologies), both following the manufacturer's recommended protocols. The RNA was purified using the MEGAclear Transcription Clean‐Up Kit (Life Technologies) and eluted in nuclease‐free water (Ambion). The RNA solutions were aliquoted and stored at −80°C until use. For microinjection, 1 µL of Cas9d^Ultra^ mRNA or 9dCBE mRNA (500 ng/µL) was mixed with 1 µL of gRNA (500 ng/µL) and 3 µL of nuclease‐free water. The mixture was centrifuged at 14 000 rpm and 4°C for 10 min, and the supernatant was transferred to a new 200‐µL RNase‐free tube for microinjection. All primer sequences used for amplifying the templates are listed in Table S9.

### 5.11 Phylogenetic Tree Construction

To construct a phylogenetic tree of representative type II orthologs [54] and IscB orthologs [21, 64], multiple sequence alignment of all proteins was performed using MAFFT‐fftnsi (v7.526) with the –maxiterate 1000 parameter [65]. Alignment columns with gap frequencies over 50% were then trimmed using trimAl (v1.5.rev0) with ‐gt 0.5 argument [66]. The processed alignment was then used to construct the phylogenetic tree using IQ‐TREE3 (v3.0.1) with 1000 ultrafast bootstrap replicates and –alrt 1000 –bnni to optimize the tree and other default parameters [67, 68].

### 5.12 Animals

To generate *Tyr*‐modified mice, female B6D2F1 (C57BL/6J × DBA2) mice (7‐9 weeks old) were superovulated by exogenous gonadotropin treatment (PMSG and hCG) and then mated with male B6D2F1 mice (10‐15 weeks old) [47]. 8‐week‐old pseudo‐pregnant female ICR mice at 0.5 dpc were used as recipients for the transfer of 2‐cell embryos. The mouse experiments were approved by the Biomedical Research Ethics Committee at the College of Animal Science and Technology, Northwest A&F University.

For *in vivo Pcsk9* editing, pregnant wild‐type C57BL/6 mice were purchased from Zhuhai Bestest Biotechnology Co., Ltd. AAV vectors were produced by PackGene Biotech. AAV9‐9dABE/9dCBE were administered via intraperitoneal (IP) injection at P3‐P5 at a dose of 5E13 vg/mouse. Control mice received an equal volume of PBS. Five weeks after injection, liver tissues and serum samples were harvested to evaluate base editing efficiency and LDL‐C levels, respectively. The mouse experiments were approved by the Animal Care and Use Committee of the Zhongshan Institute for Drug Discovery, Shanghai Institute of Materia Medica, Chinese Academy of Sciences. All mice were housed in a 12‐h light/dark cycle room with water and food ad libitum.

### 5.13 One‐Cell Zygote Microinjection and Embryo Transplantation

Microinjection and embryo transfer were performed as previously described [47]. In brief, after 21 h of human chorionic gonadotropin injection, fertilized eggs were isolated from the oviducts and cleared in M2 medium and M16 medium (supplemented with amino acids), respectively. The RNA mixture of the editing system (mRNA: 100 ng/µL; gRNA: 100 ng/µL) was injected into the cytoplasm of zygotes with well‐recognized pronuclei. Microinjection was performed within a HEPES‐CZB medium (containing 5 µg/mL cytochalasin B) droplet using a Piezo microinjector (HARIOLAB). Injected zygotes were cultured in M16 with amino acids at 37°C under 5% CO_2_ in a humidified incubator until they reached the 2‐cell stage by ∼1.5 days. Subsequently, 25 2‐cell embryos were transferred into the oviducts of pseudo‐pregnant ICR females at 0.5 dpc.

### 5.14 Embryos and Mouse Genotyping

To evaluate the editing efficiency of Cas9d^Ultra^ and 9dCBE at the *Tyr* locus in injected embryos, individual two‐cell stage embryos or blastocysts were lysed in 2 µL of lysis buffer to release genomic DNA. For genotyping *Tyr*‐modified offspring, tail tissues were collected from neonatal pups at postnatal day 7. Genomic DNA was extracted using a tissue lysis kit (Vazyme) according to the manufacturer's instructions. The targeted regions in embryos and offspring mice were amplified by two‐round PCR using 2×Taq mix (Vazyme), followed by the addition of barcodes and Illumina adapters. The resulting libraries were subjected to high‐throughput sequencing. Editing efficiencies and genotypes resulting from Cas9d^Ultra^ and 9dCBE were analyzed using CRISPResso2 (v2.3.1). The Sanger sequencing data were analyzed using EditR (v1.0.10) (https://moriaritylab.shinyapps.io/editr_v10) [69].

### 5.15 In Vivo Editing Efficiency and Serum Analysis

To evaluate editing efficiency, whole liver tissues were collected for genomic DNA isolation. The target *Pcsk9* locus was amplified and barcoded by two rounds of PCR. HTS data were analyzed using CRISPResso2. For serum LDL‐C quantification, blood samples were collected and centrifuged at 3000 rpm for 15 min at 4°C to separate the serum, and then stored at −80°C. LDL‐C levels were measured using an LDL‐C detection kit (Sangon Biotech, #D799859) following the manufacturer's protocol.

### 5.16 Statistical Analysis

All values are presented as mean ± standard deviation (s.d.), except for the editing window measurements of base editors and editing efficiency of *Tyr* mice, which are exhibited as mean ± standard error of the mean (s.e.m.). Detailed descriptions of the statistical methods used throughout the manuscript are denoted in the corresponding figure legends. For comparisons between two independent groups, a two‐tailed, unpaired Student's *t*‐test was used. One‐way ANOVA was used for multiple comparisons. A *p*‐value < 0.05 was considered statistically significant. The experiments were not randomized and the researchers were not blinded to allocation during experiments and outcome assessment. Statistical analyses were performed using GraphPad Prism (version 8.2.1) (www.graphpad.com).

## Author Contributions

Q.X. conceived the project. Q.X., Z.G., Y.W., and G.L. jointly supervised the research. Q.X. designed experiments and participated in their execution and analyzed experiments. Q.X. and Z.T. conducted plasmid construction, endogenous sites and off‐target sites amplification for NGS, and R‐loop assay. Z.T. and Q.X. performed cell culture and transfection. Z.T., L.W., and J.L. jointly carried out FACS analyses and cell sorting. L.W. and J.L. assisted with plasmid cloning and extraction. K.X., S.L., and Q.Z. performed IVT of gRNA, mRNA, mouse embryo microinjection and transplantation. Z.W., Q.X., L.W., and S.L. conducted GUIDE‐seq, and Z.W. analyzed GUIDE‐seq data. Q.X. conducted mouse genotyping, bioinformatic analyses, data analysis, and figure organization. D.H. designed and constructed the PAM library and R‐loop plasmids, identified PAMs in *E. coli*. G.L. conducted in vivo experiments and LDL‐C measurement. X.Z. participated in discussing the experiments. Q.X., D.H., Z.G., Y.W. and G.L. wrote the manuscript with data contributed by all authors.

## Conflicts of Interest

A patent application (#ZL202610251120.1) related to this work has been filed by Jinhua Institute of Zhejiang University, with Z.G., Q.X. and Z.T. as co‐inventors. Z.G. is a co‐founder of Zenomics Inc., Zcapsule Inc. and µZen Inc. The remaining authors declare no other competing interests.

## Supporting information




**Supporting File 1**: advs77279‐sup‐0001‐SuppMat.pdf.

**Supporting File 2**: advs77279‐sup‐0002‐SuppMat.xlsx.

## Data Availability

The data that support the findings of this study are available from the corresponding author upon reasonable request.
