# Supplementary material for: Compact 9dBEs Enable Efficient and Precise Genome Editing in Mammalian Cells and In Vivo
Source: Adv Sci (Weinh). 2026 Aug 19:e77279. Online ahead of print. doi: 10.1002/advs.77279 (PMC13487866; doi:10.1002/advs.77279)
Supplement: Supplementary file 1 [file ADVS-9999-e77279-s002.pdf]

# Supporting Information

## Compact 9dBEs enable efficient and precise genome editing in mammalian cells and *in vivo*

Qingquan Xiao[1,*], Zhijin Tian[1], Luqi Weng[1], Kun Xu[2,3], Dingyi Han[4], Zhaowei Wu[5], Junnan Li[6], Shangpu Li[2], Qijing Zhang[2], Xiaohui Zhao[1], Guoling Li[7,*], Yinghui Wei[2,3,*], Zhen Gu[1,*]

## Supplementary figures

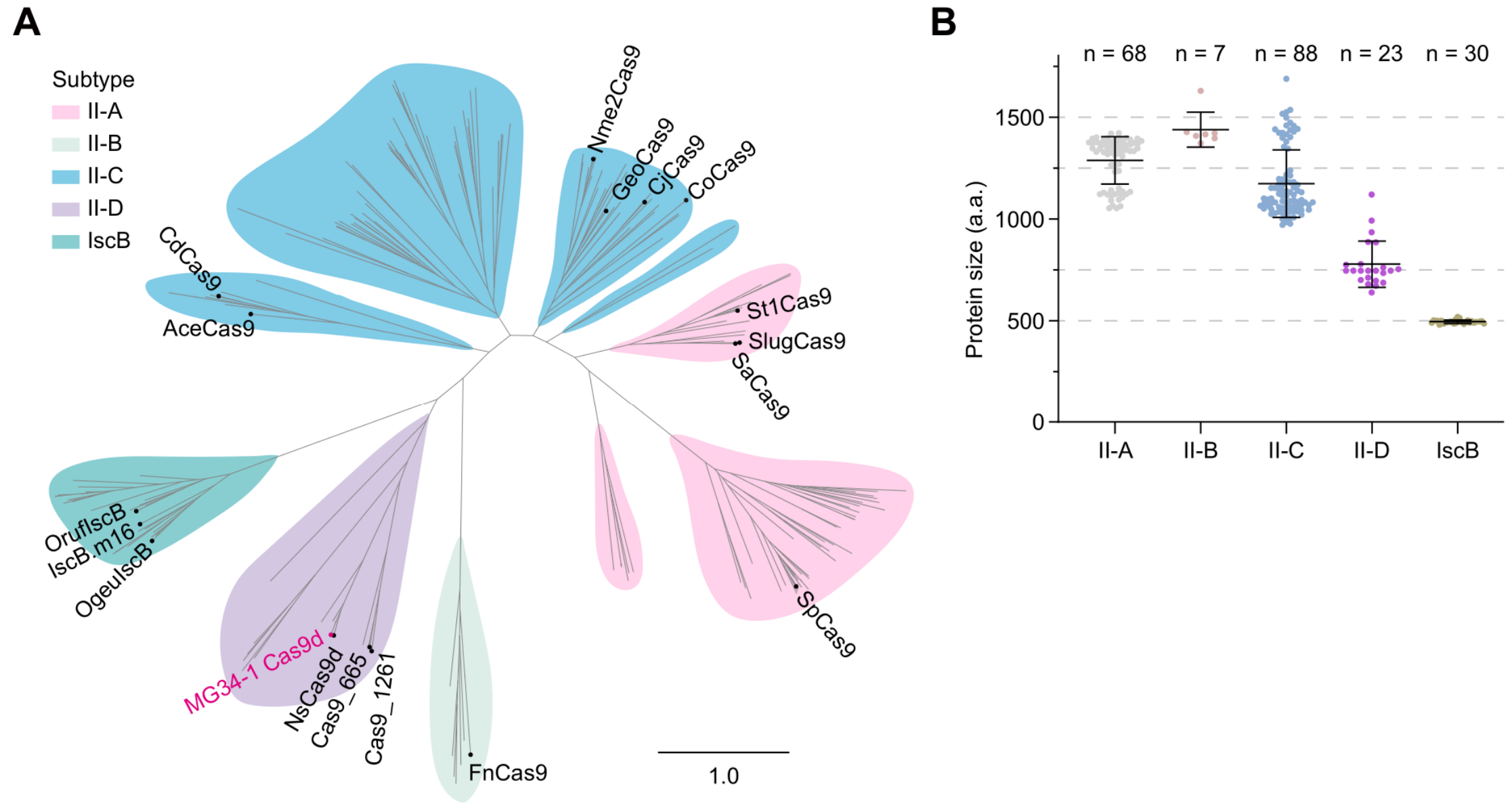


**Figure S1** | Phylogenetic diversity and effector size distribution of type II CRISPR systems. (A) Phylogenetic tree of representative Cas9 orthologs from four type II subtypes and IscB systems. Subtypes are distinguished using different colors. Representative Cas9s and IscBs are highlighted with dots. (B) Size distribution of the proteins shown in (A). Type II-D Cas9 (referred to as Cas9d) proteins are significantly smaller (average size approximately 750 aa) compared to other subtypes. Dots indicate individual protein sizes. Data are shown as mean ± s.d.

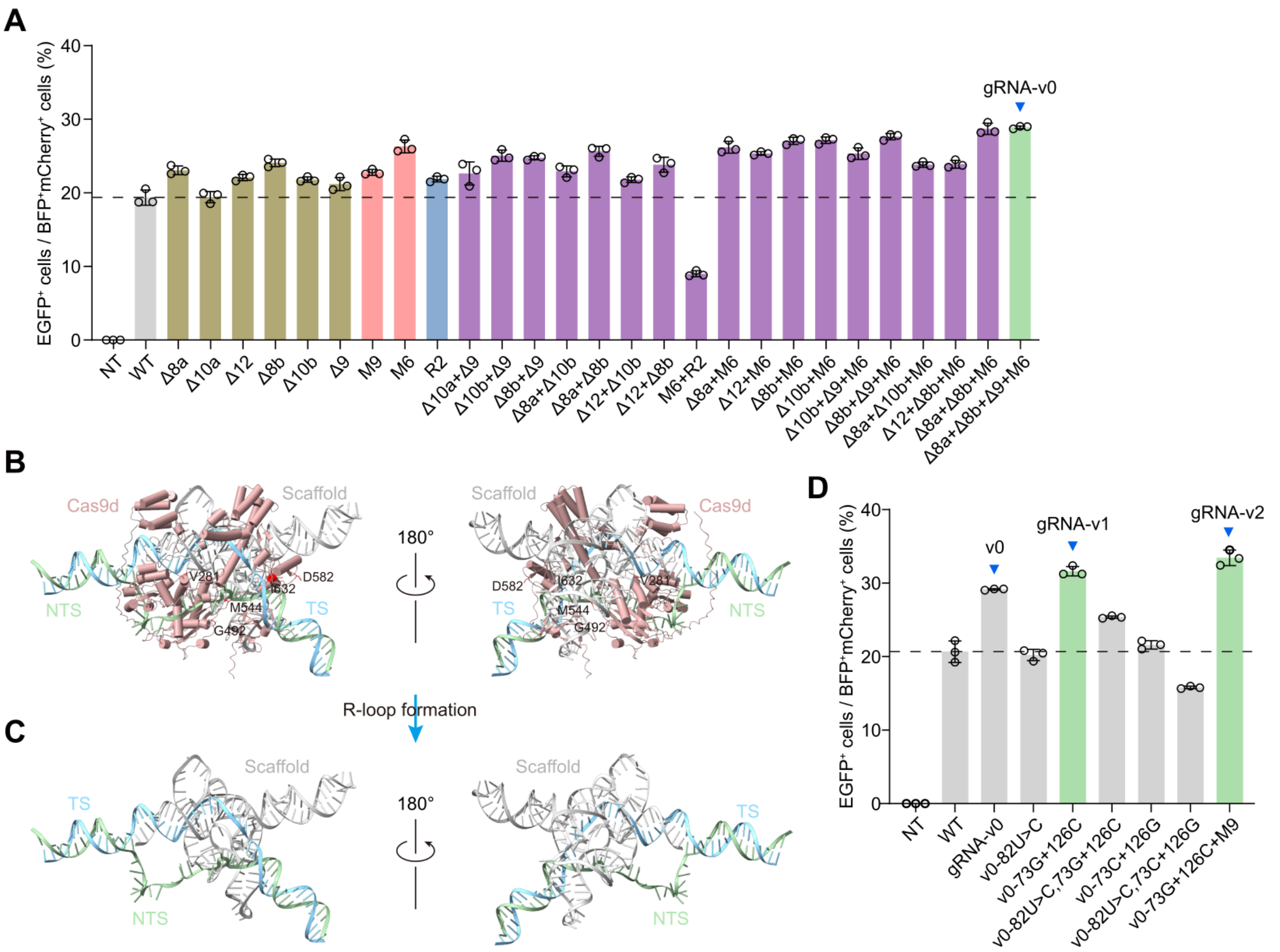


**Figure S2** | Structure-guided gRNA optimization improves Cas9d editing activity. (A) Editing efficiency of Cas9d using truncated or mutated gRNA scaffolds compared to that using the wild-type scaffold. (B) Overall structure of the Cas9d-gRNA-dsDNA ternary complex predicted by AlphaFold3. Key residues highlighted in red. (C) Detailed view of R-loop formation within the gRNA-dsDNA heteroduplex region of the complex. (D) Rational combination of gRNA-v0 with beneficial mutations improved Cas9d cutting efficiency. Data are shown as mean ± s.d. (n = 3 independent biological replicates).

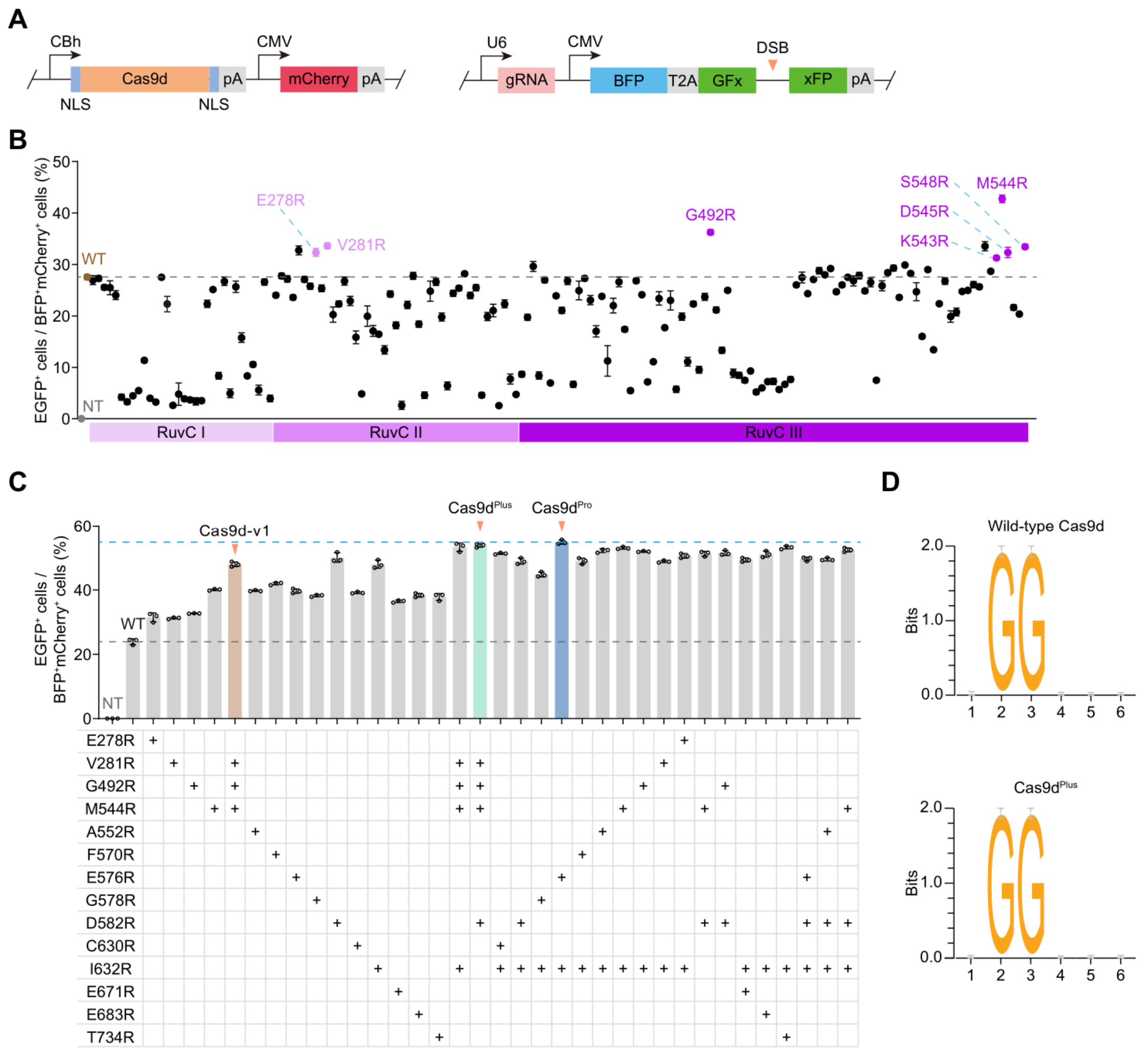


**Figure S3** | Protein engineering enhances Cas9d activity. (A) Diagram illustrating the dual-plasmid system applied for Cas9d variant screening. (B) Editing efficiencies of arginine substitutions in Cas9d three RuvC nuclease domains. NT means non-target guide. WT denotes wild-type Cas9d system. Each point represents the average editing efficiency of n = 3 independent biological replicates for a given variant. Data are shown as mean ± s.d. (C) Combination of beneficial mutations resulting in largely enhanced Cas9d activity. Data are displayed as mean ± s.d. (n = 3). (D) WebLogo profiles illustrating the PAM recognition by wild-type Cas9d and Cas9d$^{Plus}$, characterized by *E. coli* plasmid depletion assay.

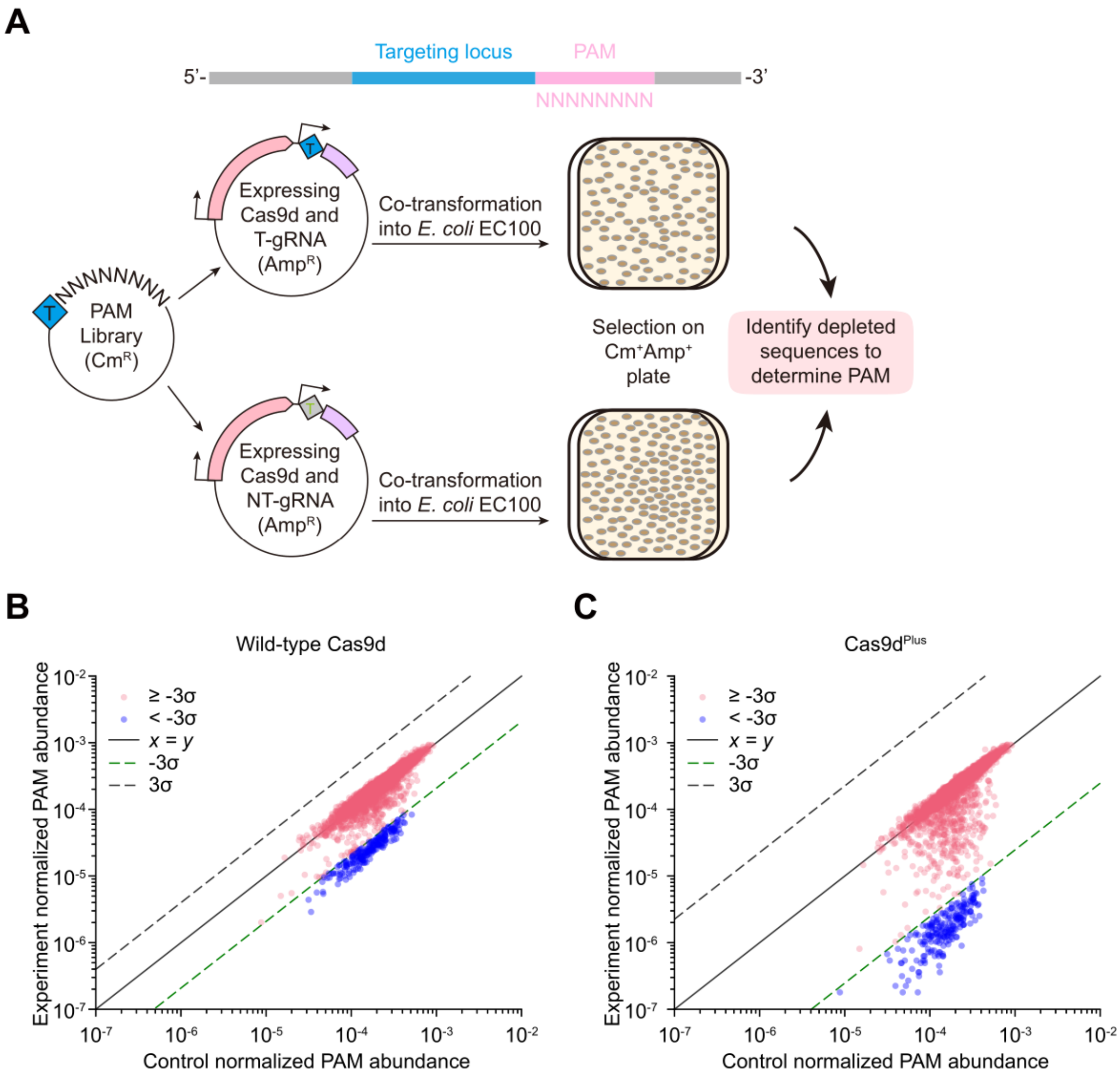


**Figure S4** | Characterization of PAM preferences by plasmid depletion. (A) Diagram illustrating the dual-plasmid system used for the Cas9d PAM depletion assay. (B and C) Scatter plots displaying the distribution of PAM depletion data for wild-type Cas9d (B) and $\text{Cas9d}^{\text{Plus}}$ (C). Diagonal lines represent a ratio of 1 between the normalized PAM abundance in the experimental versus control groups. σ means standard deviation (STD) of the distribution. Pink dots indicate PAMs with a ratio ≥ -3σ, while blue dots denote PAMs with a ratio < -3σ.

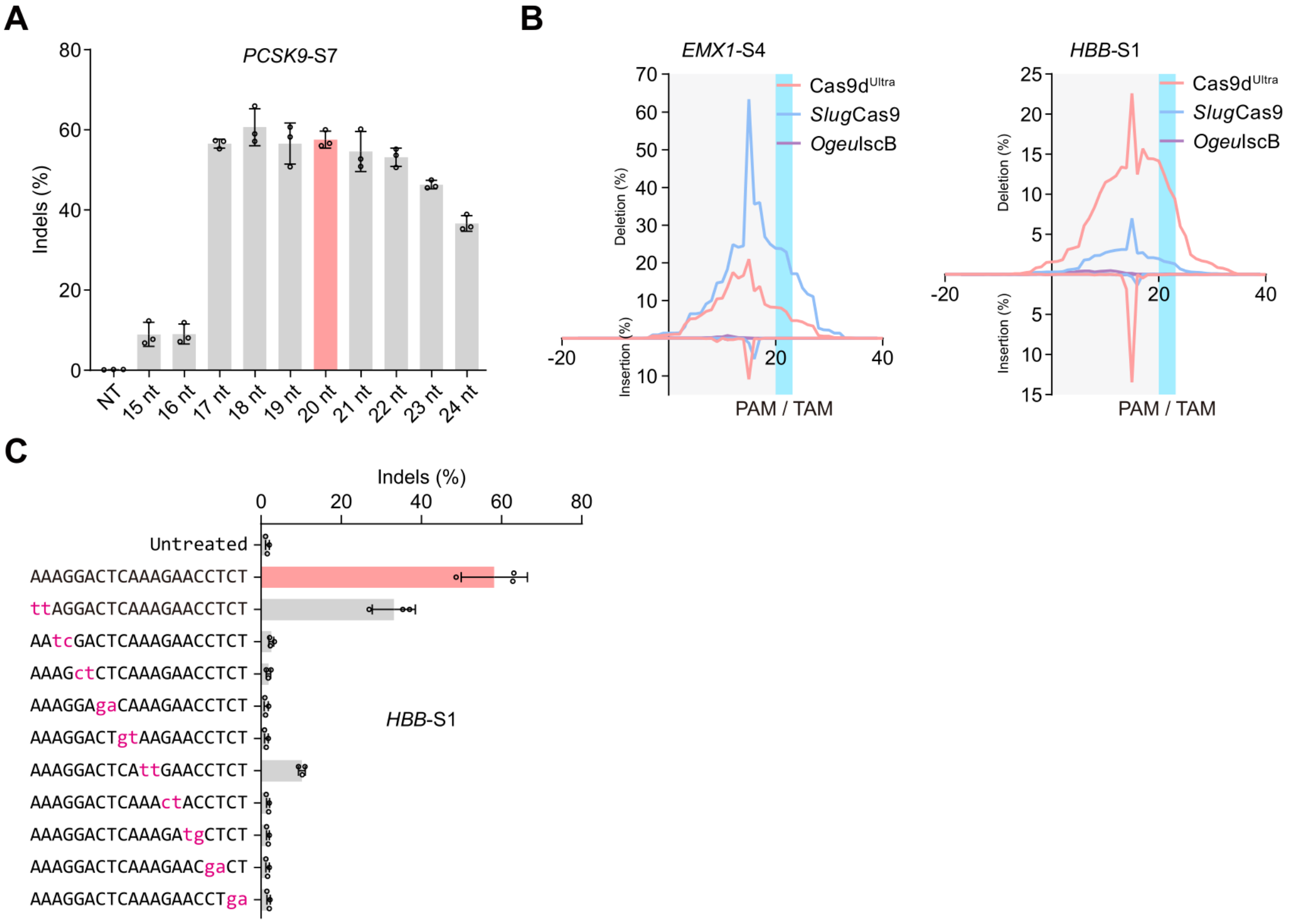


**Figure S5** | Cas9d$^{\text{Ultra}}$ cleavage characteristics at endogenous loci. (A) Histogram showing Cas9d$^{\text{Ultra}}$ cleavage efficiency with different spacer lengths at the *PCSK9*-S7 site in HEK293T cells. (B) Indel patterns generated by Cas9d$^{\text{Ultra}}$, *Slug*Cas9 and *Ogeu*IscB at *EMX1*-S4 and *HBB*-S1 sites. (C) Mismatch tolerance of Cas9d$^{\text{Ultra}}$ within the spacer at *HBB*-S1 locus. Data in (A) and (C) are represented as mean ± s.d. (n = 3 independent biological replicates).

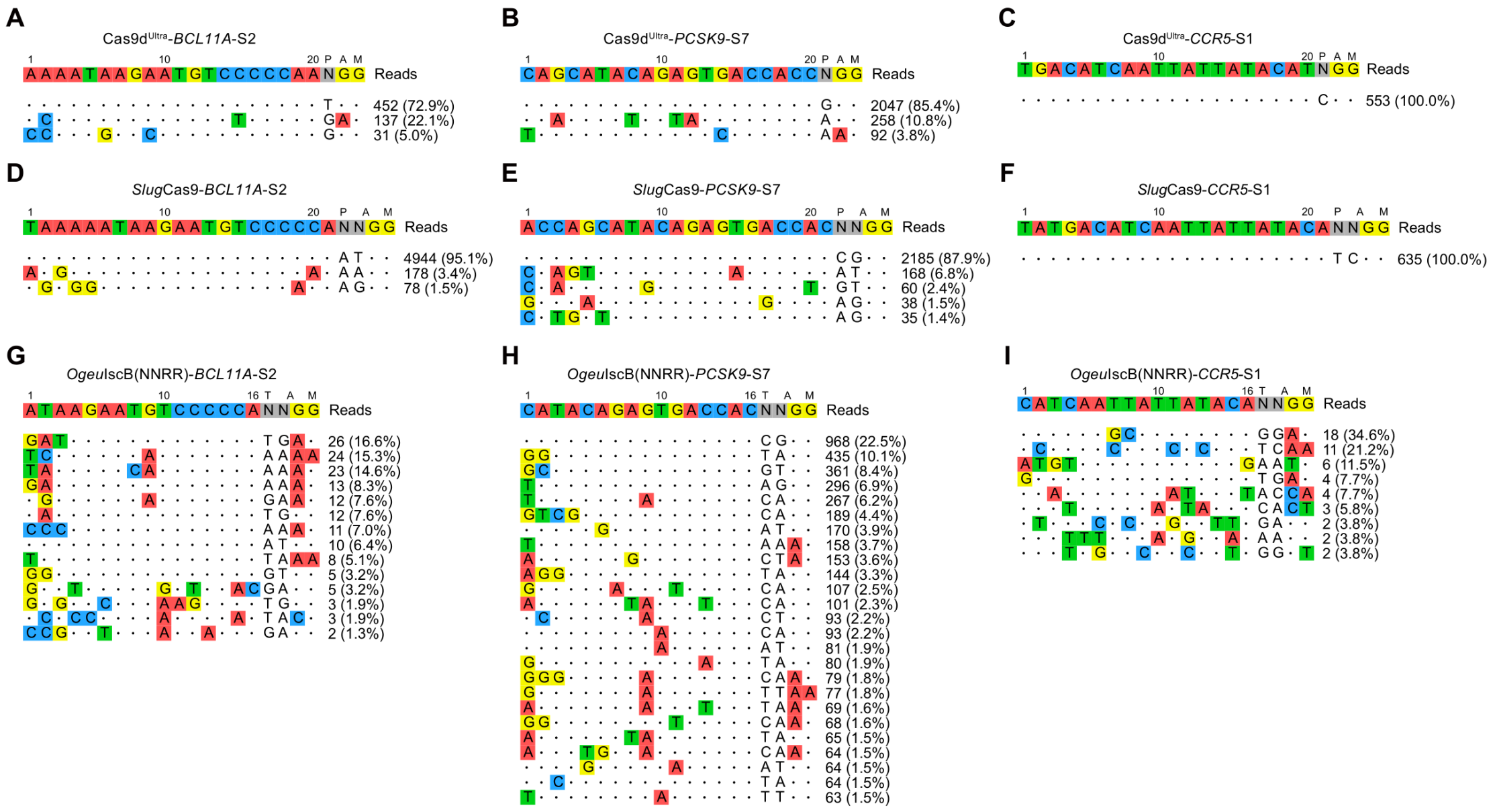


**Figure S6** | Genome-wide specificity of the Cas9d$^{Ultra}$ system. (A-C) Off-target loci identified by GUIDE-seq for Cas9d$^{Ultra}$ at *BCL11A*-S2 (A; 2 off-targets), *PCSK9*-S7 (B; 2 off-targets) and *CCR5*-S1 (C; no off-targets). (D-F) GUIDE-seq-identified off-target sites for *Slug*Cas9 at *BCL11A*-S2 (D; 2 off-targets), *PCSK9*-S7 (E; 4 off-targets) and *CCR5*-S1 (F; no off-targets). (G-I) Off-target loci identified by GUIDE-seq for *Ogeu*IscB at *BCL11A*-S2 (G; 13 off-targets), *PCSK9*-S7 (H; 24 off-targets) and *CCR5*-S1 (I; 9 off-targets). On-target sequences are indicated by dotted lines. Mismatched bases in off-target sequences are color-highlighted. Sites with reads ≥ 1% and ≤ 6 mismatches were included.

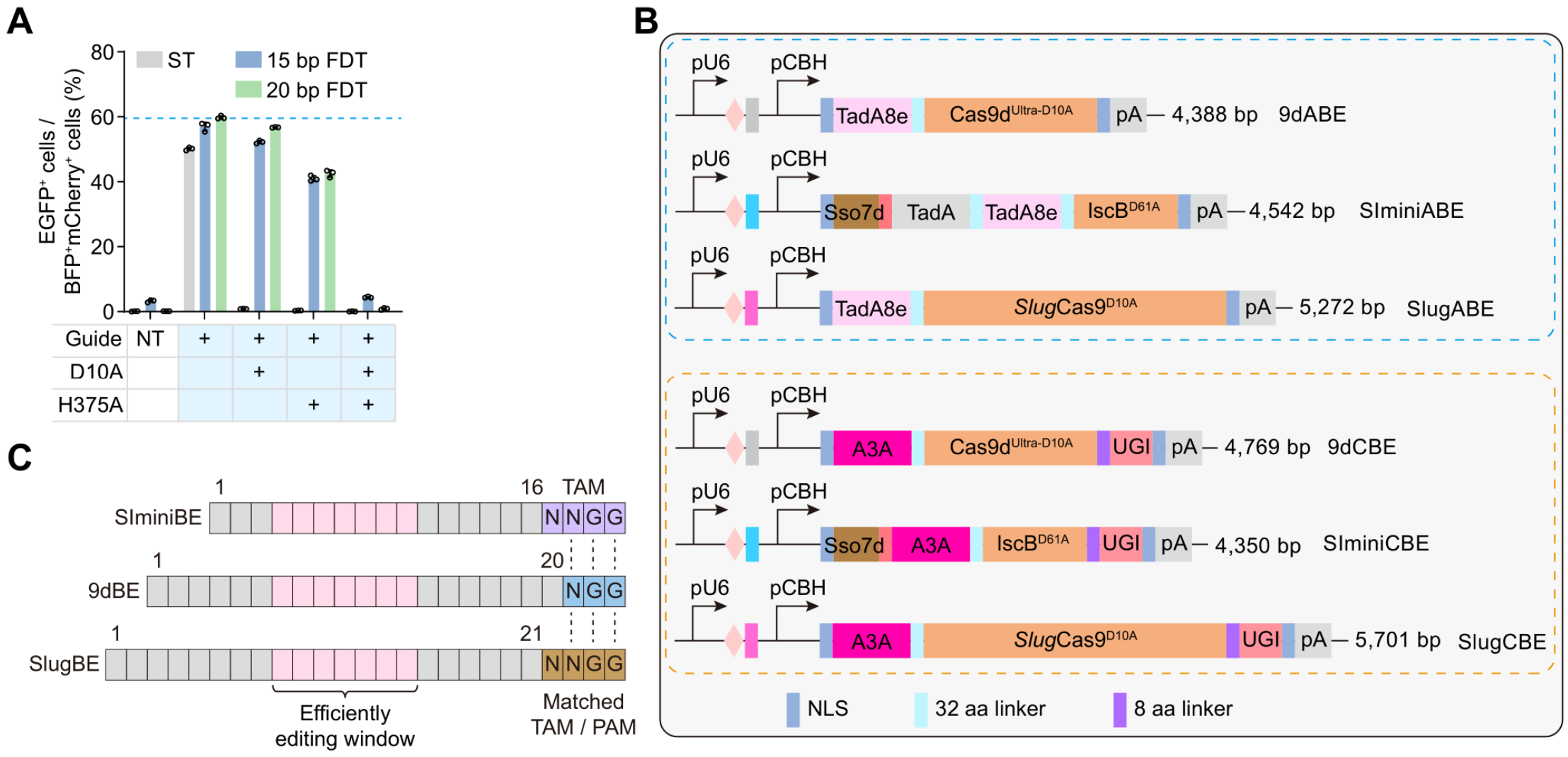


**Figure S7** | Development of Cas9d$^{Ultra}$-derived nickases and base editors. (A) Nickases activity of Cas9d$^{Ultra}$ variants (D10A and/or H375A) using a dual-target reporter system. ST, single target reporter; FDT, flanked dual-target reporter. Data are presented as mean ± s.d. n = 3 independent biological replicates. (B) Architectural schematics of ABE and CBE constructs. NLS, nuclear localization signals. (C) Overview of PAM/TAM-matched sites used to compare 9dBEs, SlugBEs and SIminiBEs.

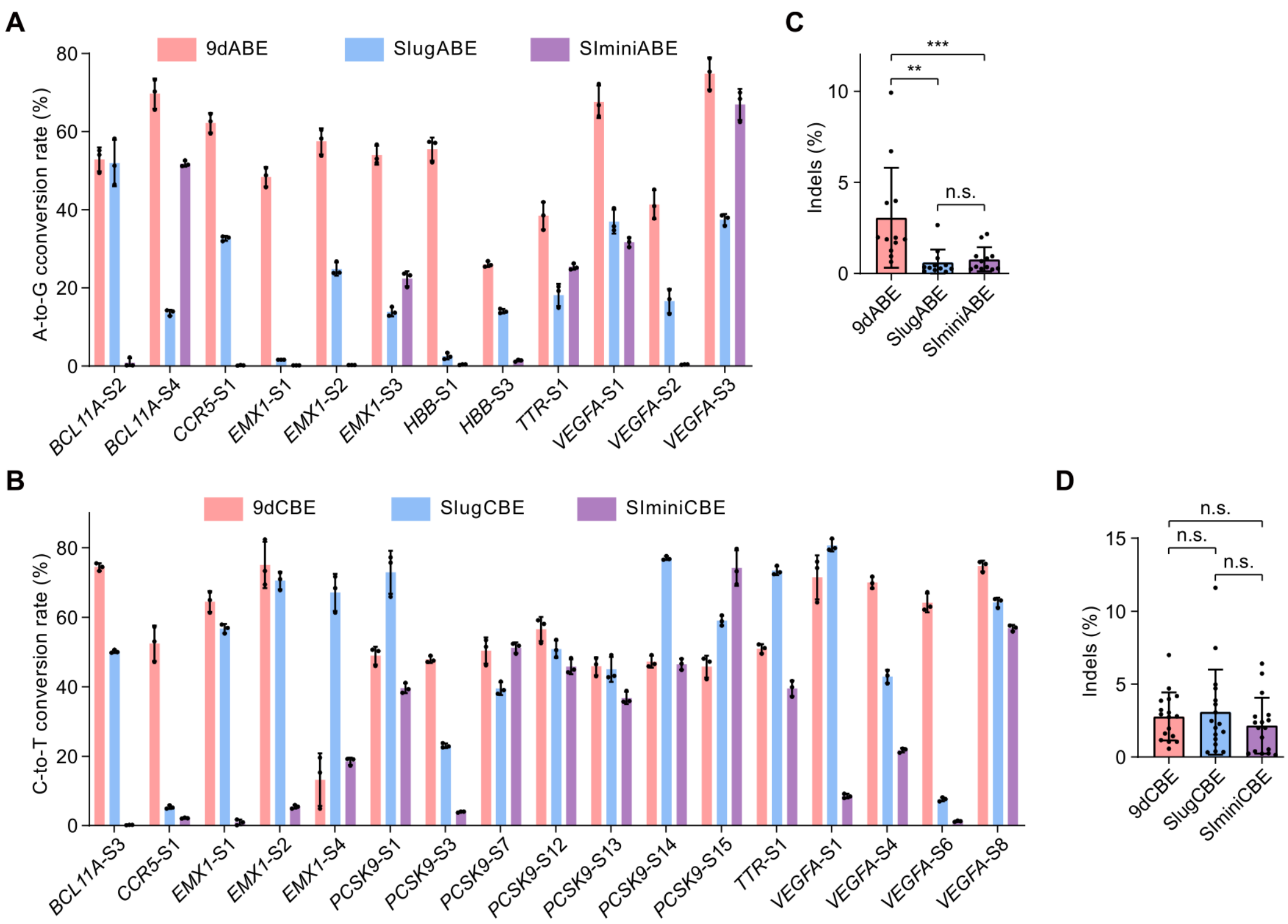


**Figure S8** | Base editing efficiency and indels of ABE and CBE systems. (A and B) The optimal editing efficiency at each given genomic target of ABE (A) and CBE (B) systems in HEK293T cells. n = 3 independent biological replicates. (C and D) Indels generated by ABEs (C) at 12 sites, CBEs (D) at 17 sites. Data are presented as mean ± s.d. To compare the means, Kruskal-Wallis test was used. $^{**}P < 0.01$; $^{***}P < 0.001$. n.s. denotes non-significant.

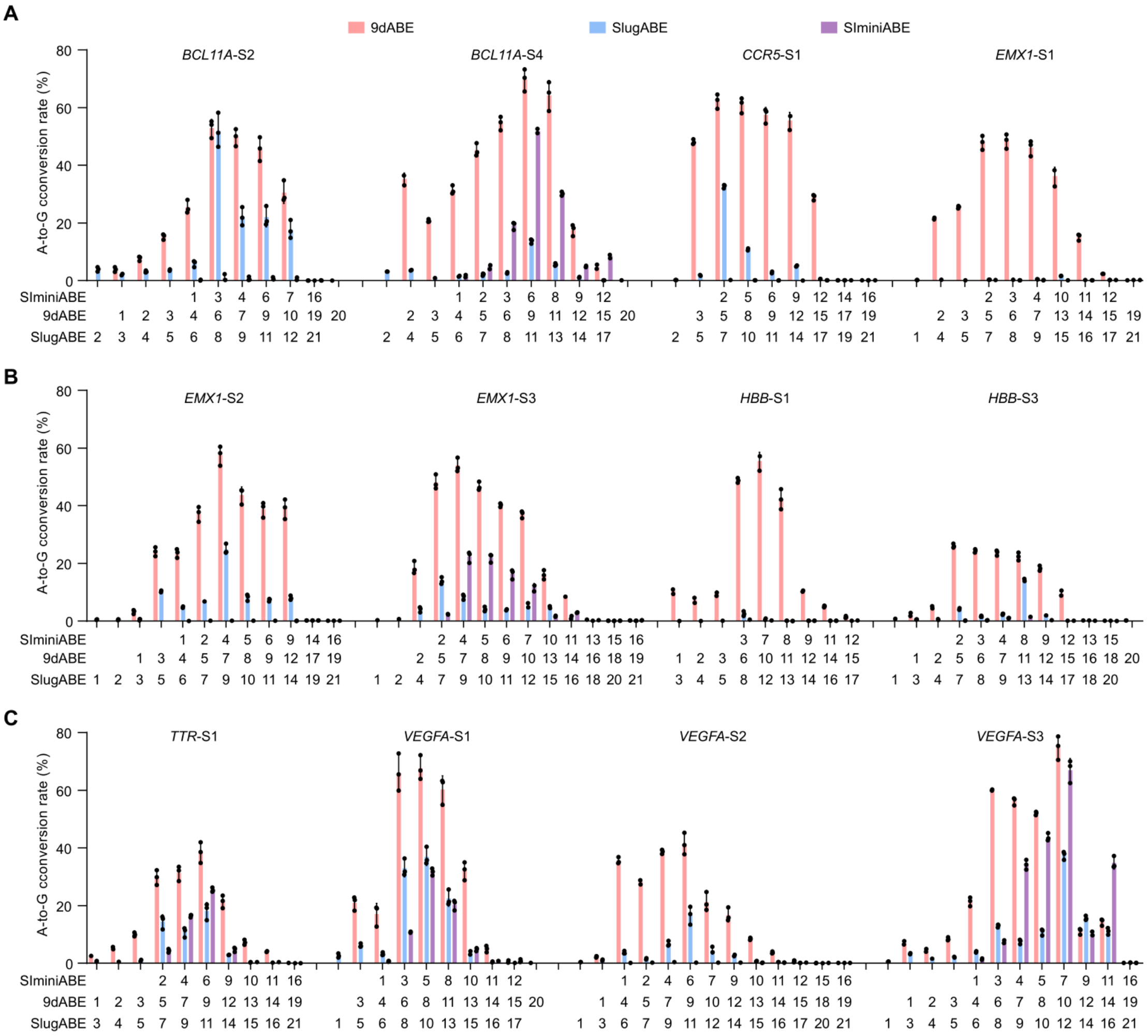


**Figure S9** | ABE-mediated A:T-to-G:C base conversions. (A-C) Base editing efficiencies of 9dABE, SlugABE and SlminiABE at each A position across 12 sites. Data are presented as mean ± s.d. n = 3 independent biological replicates.

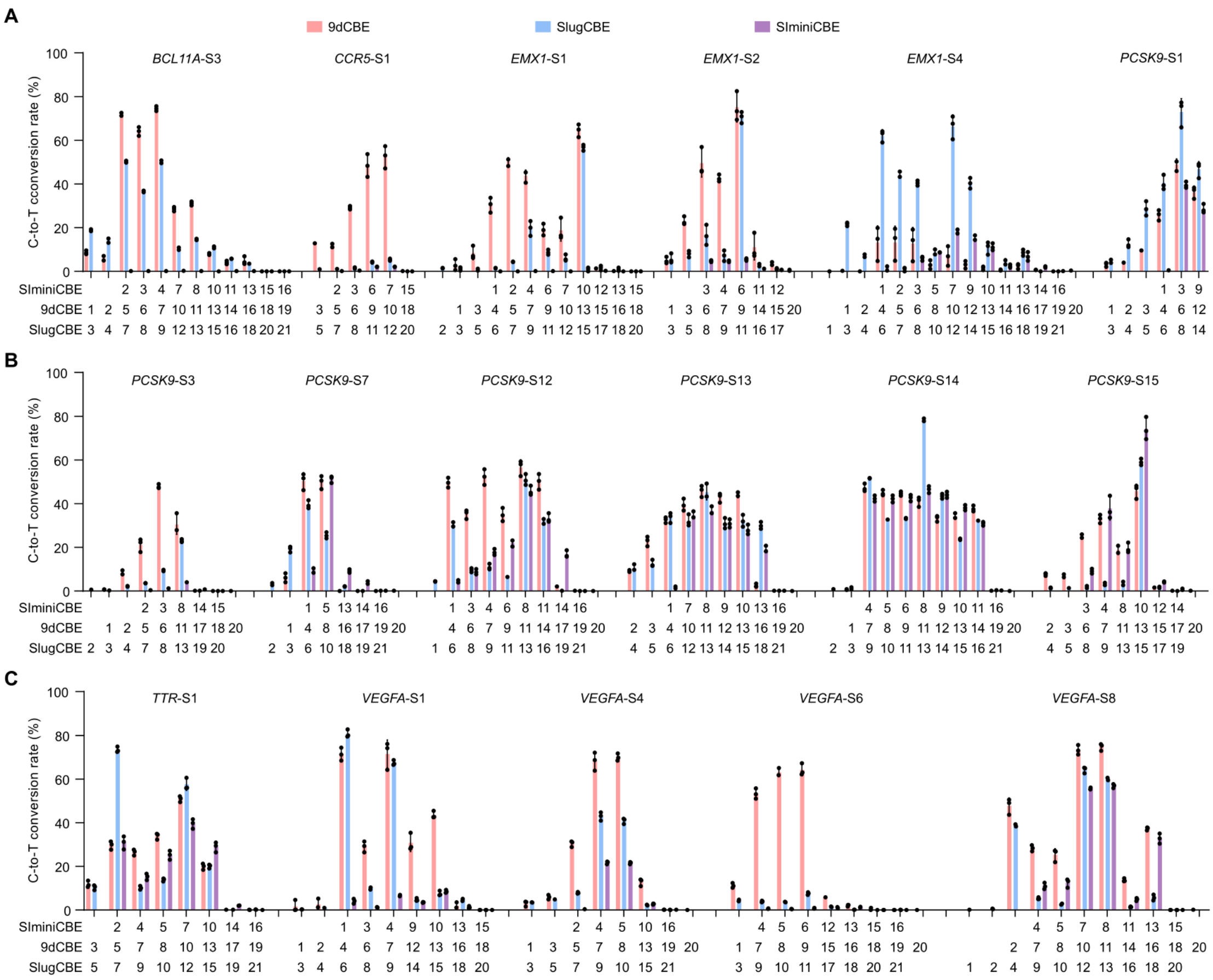


**Figure S10** | CBE-mediated C:G-to-T:A base conversions. (A-C) Base editing efficiencies of 9dCBE, SlugCBE and SIminiCBE at each C position across 17 sites. Data are presented as mean ± s.d. n = 3 independent biological replicates.

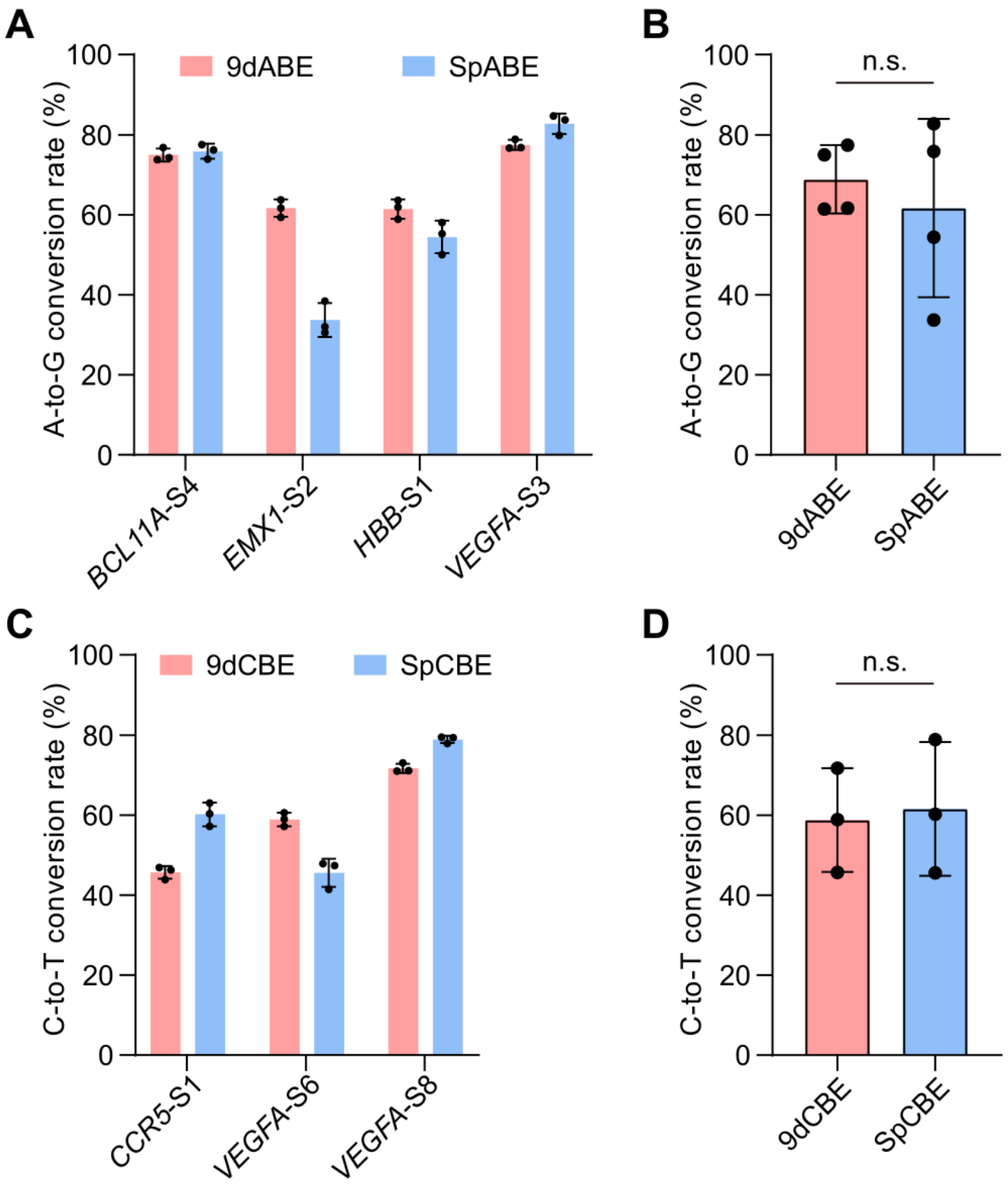


**Figure S11** | Base editing efficiencies of 9dBEs and SpBEs. (A) A-to-G editing efficiencies of 9dABE and SpABE at four human sites. (B) Statistical analysis of the average A-to-G editing efficiencies shown in (A). (C) C-to-T editing efficiencies of 9dCBE and SpCBE at three endogenous sites. (D) Statistical analysis of the average C-to-T editing efficiencies shown in (C). Data are presented as mean ± s.d. n = 3 independent biological replicates. To compare the means, an unpaired two-tailed Student's *t*-test was used in (B and D). n.s. denotes non-significant.

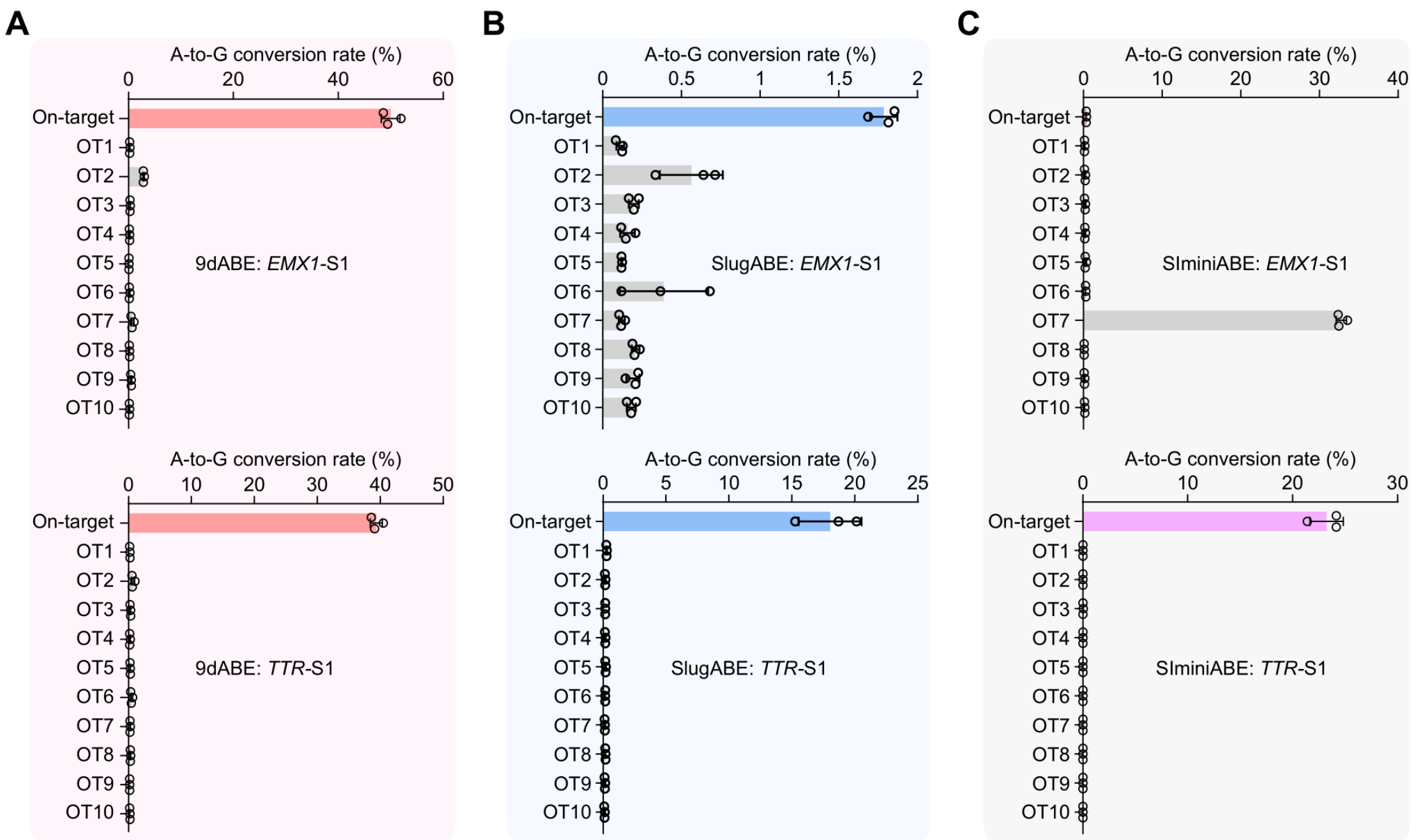


**Figure S12** | Evaluation of gRNA-dependent off-target effects in ABE systems. (A to C) Targeted deep-sequencing assessment of off-target effects for 9dABE (A), SlugABE (B) and SIminiABE (C) in HEK293T cells at Cas-OFFinder-predicted sites. Values and error bars reflect the mean ± s.d. of n = 3 independent biological replicates.

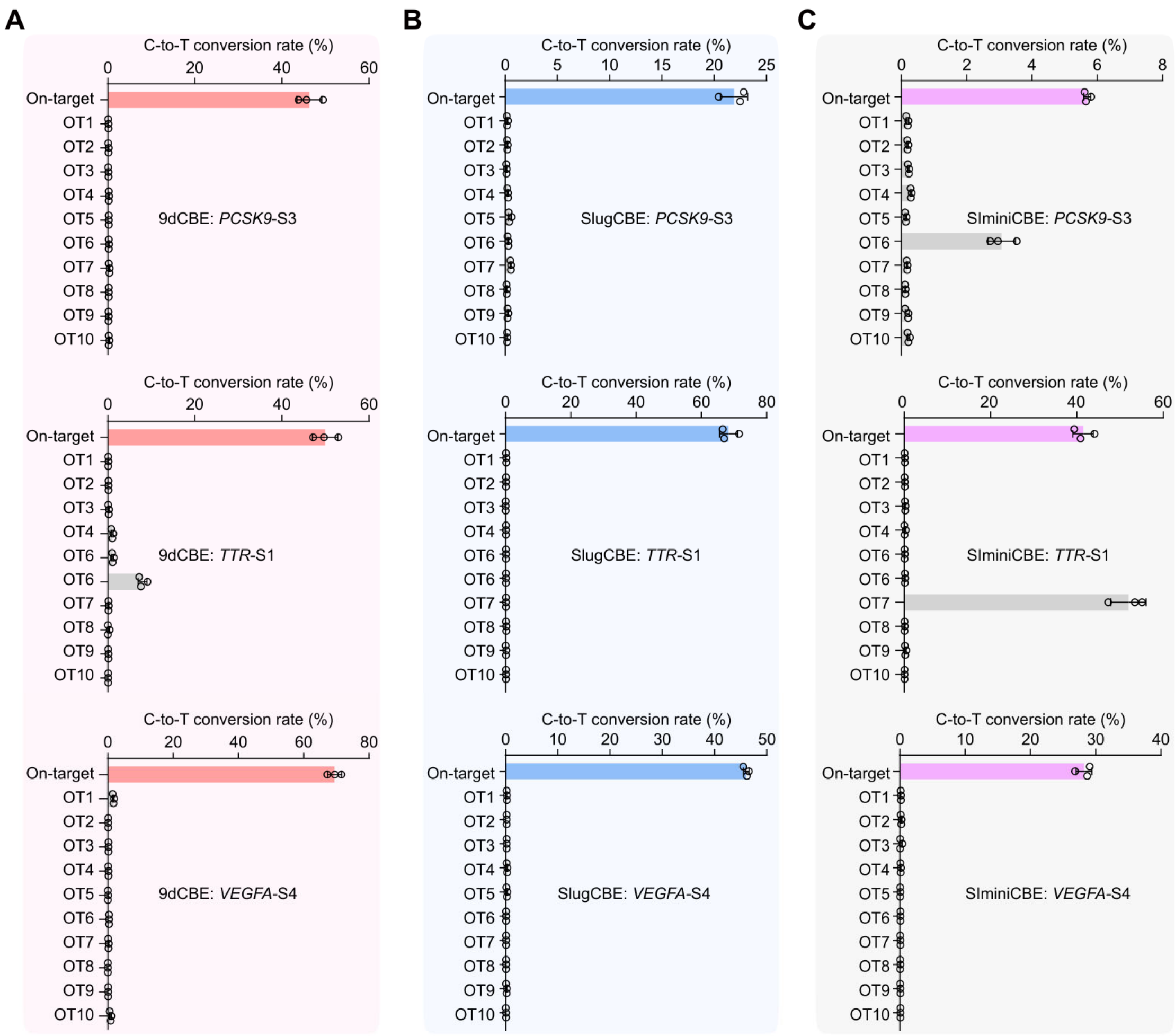


**Figure S13** | Detection of gRNA-dependent off-target effects in CBE systems. (A to C) Off-target effects analysis by targeted deep-sequencing for 9dCBE (A), SlugCBE (B) and SIminiCBE (C) at Cas-OFFinder-predicted sites in HEK293T cells. Data are represented as mean ± s.d. (n = 3 independent biological replicates).

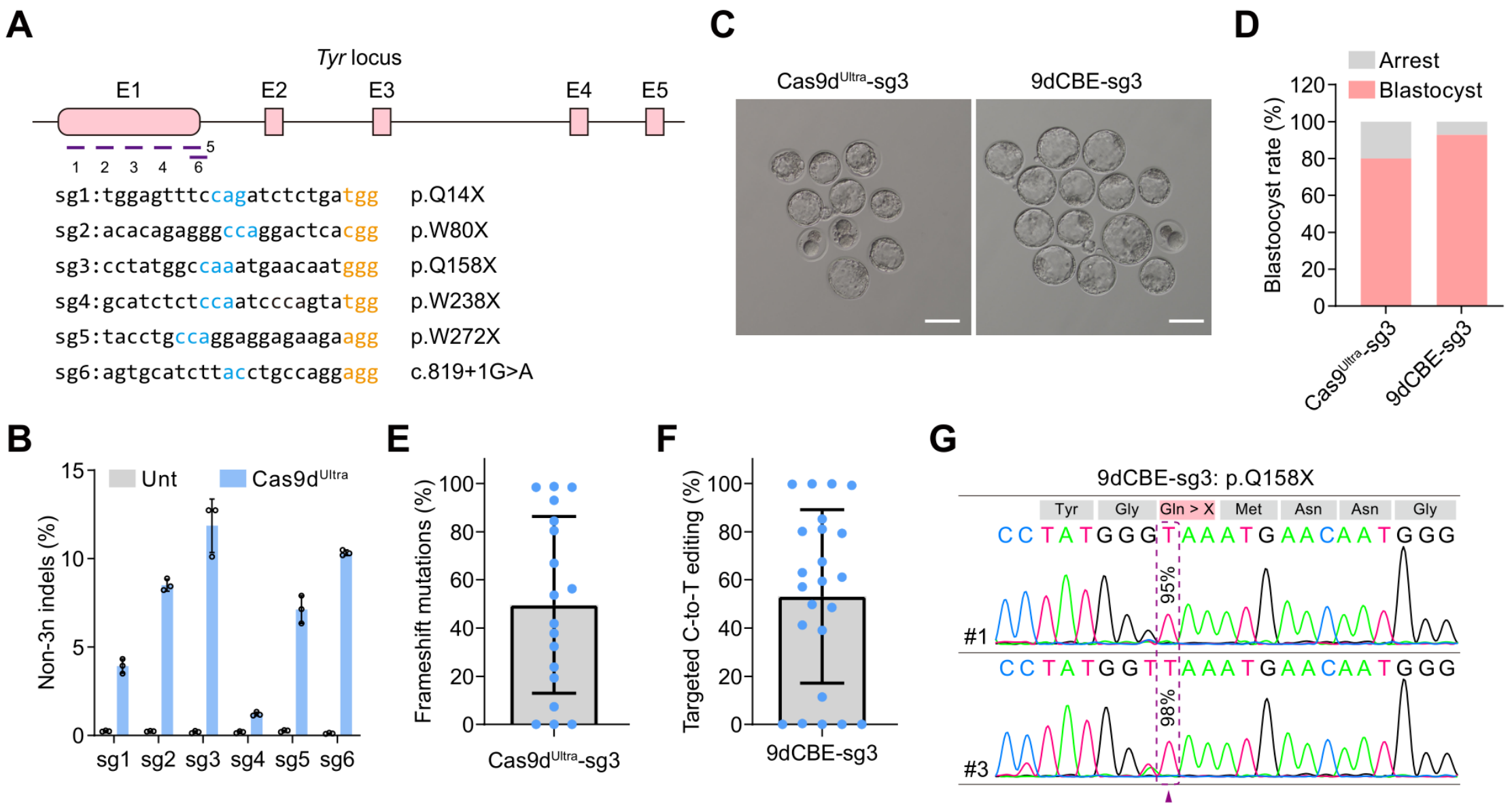


**Figure S14** | Cas9d$^{\text{Ultra}}$ and 9dCBE mediated efficiently editing *Tyr* locus in mouse embryos. (A) Schematic delineating the mouse *Tyr* locus and experimental design of six guides targeting the exon 1 to induce premature stop codons or disrupt splicing. Target bases and PAMs are highlighted in cyan and orange, respectively. (B) Frameshift mutation (non-3n indels) efficiency generated by Cas9d$^{\text{Ultra}}$ at six *Tyr* target loci in N2a cells (n = 3 independent biological replicates). (C) Blastocysts derived from mouse zygotes microinjected with Cas9d$^{\text{Ultra}}$-sg3 or 9dCBE-sg3 targeting the *Tyr* gene. Scale bar, 100 μm. (D) Blastocyst development rates from (C). (E and F) Editing efficiencies of Cas9d$^{\text{Ultra}}$-sg3 (E; n = 18 embryos) and 9dCBE-sg3 (F; n = 23 embryos) in mouse embryos. (G) Representative Sanger sequencing chromatograms showing 9dCBE robustly induced premature stop codons (TAA) at the *Tyr* locus. Data in (B, E and F) are presented as mean ± s.d.

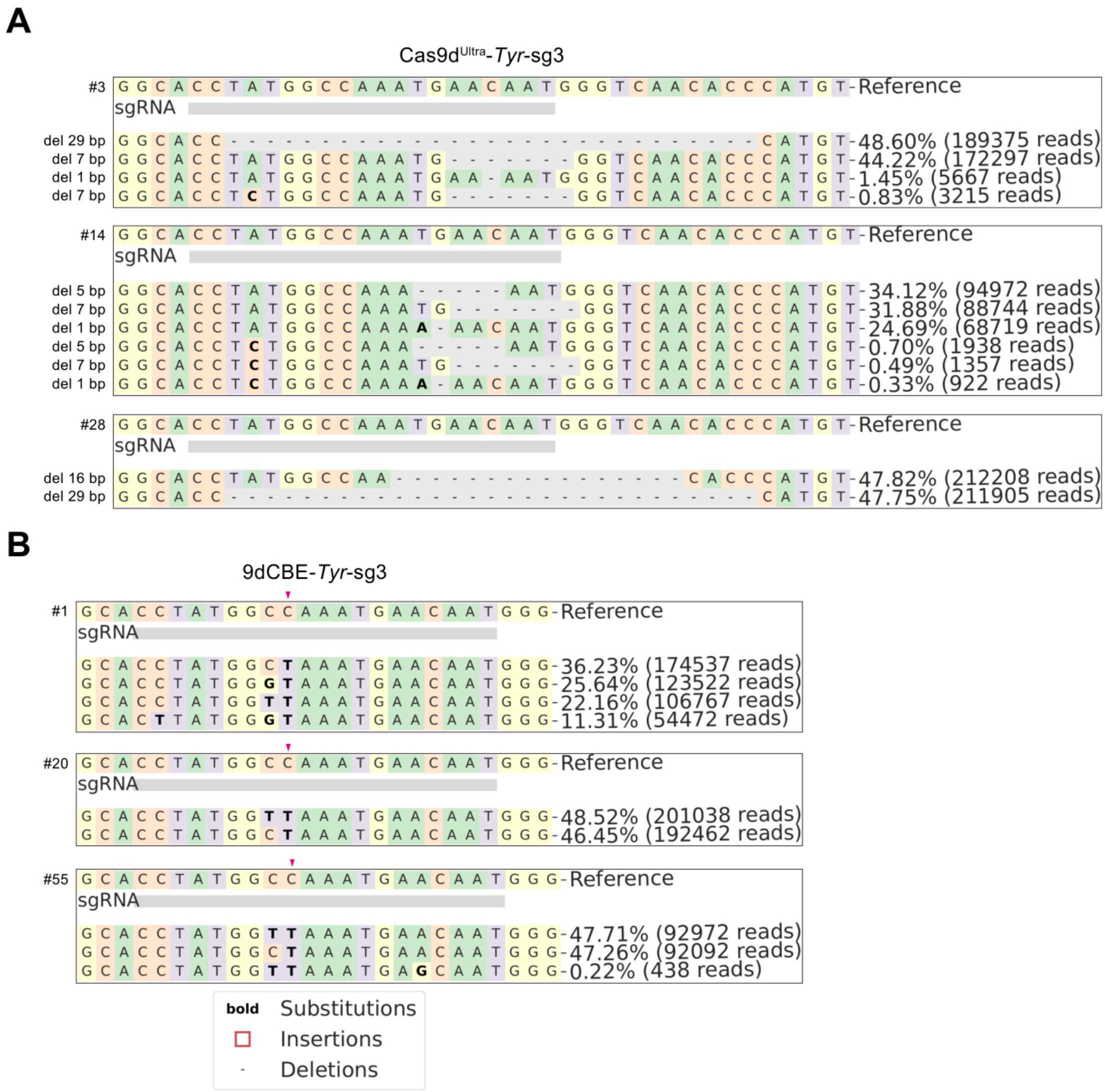


**Figure S15** | Genotyping of representative F0 mice produced by Cas9d$^{Ultra}$ and 9dCBE. (A) Sequencing profiles of three representative Cas9d$^{Ultra}$-sg3 F0 pups. Deletions (predominantly frameshift mutations) are displayed as dashed lines. (B) Three 9dCBE-sg3 F0 mouse genotypes. Targeted cytosines are indicated by red arrowheads.

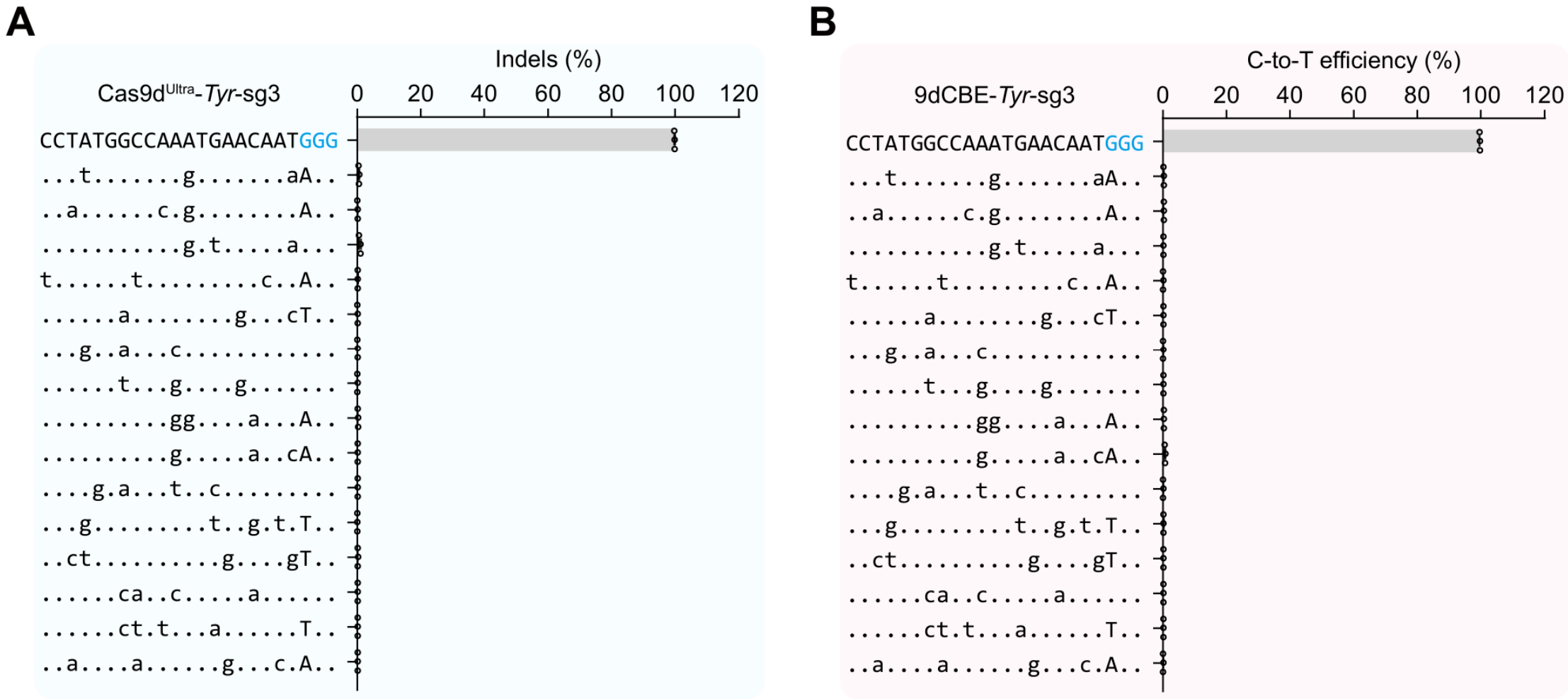


**Figure S16** | *In vivo* specificity assessment of Cas9d$^{Ultra}$ and 9dCBE. (A and B) gRNA-dependent off-target analysis at Cas-OFFinder predicted sites for Cas9d$^{Ultra}$ (A) and 9dCBE (B) by targeted deep-sequencing. Data are represented as mean ± s.d. (n = 3 mice in each group).

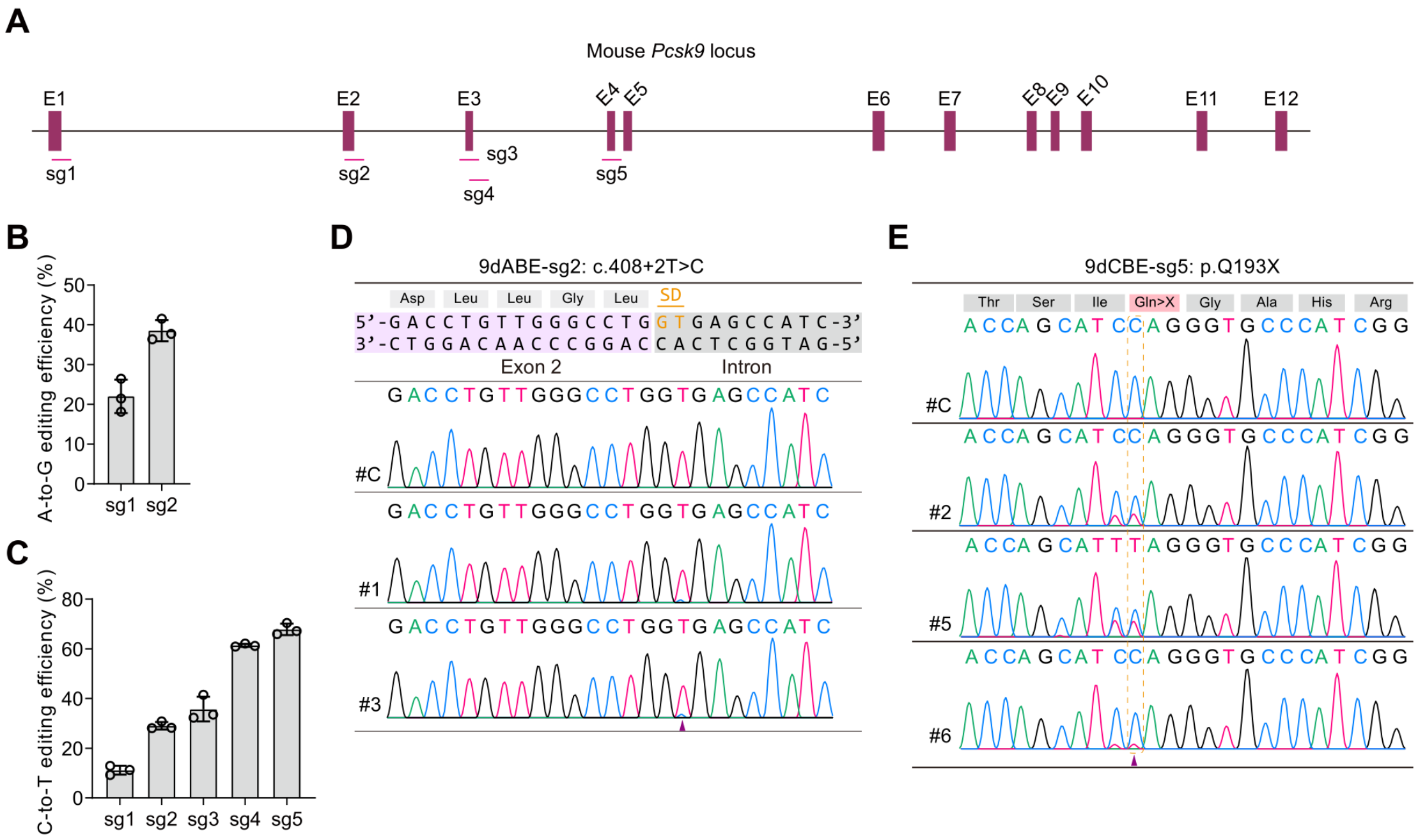


**Figure S17** | 9dBEs mediated efficiently base editing at mouse *Pcsk9* locus. (A) Schematic representation of the mouse *Pcsk9* locus. Target sites are shown in red lines. (B and C) Editing efficiency of 9dABE (B) and 9dCBE (C) in N2a cells. Data are represented as mean ± s.d. (n = 3). (D and E) Sanger sequencing trace data confirmed *in vivo* base editing of 9dABE (D) and 9dCBE (E). Purple triangles indicate the target bases. #C indicates control.
